# Enhancement of Anti-Inflammatory Activity of *Aloe vera* Adventitious Root Extracts through the Alteration of Primary and Secondary Metabolites via Salicylic Acid Elicitation

**DOI:** 10.1371/journal.pone.0082479

**Published:** 2013-12-16

**Authors:** Yun Sun Lee, Hyun Kyoung Ju, Yeon Jeong Kim, Tae-Gyu Lim, Md Romij Uddin, Yeon Bok Kim, Jin Hong Baek, Sung Won Kwon, Ki Won Lee, Hak Soo Seo, Sang Un Park, Tae-Jin Yang

**Affiliations:** 1 Department of Plant Science, Plant Genomics and Breeding Institute, and Research Institute for Agriculture and Life Sciences, College of Agriculture and Life Sciences, Seoul National University, Seoul, Republic of Korea; 2 College of Pharmacy and Research Institute of Pharmaceutical Sciences, Seoul National University, Seoul, Republic of Korea; 3 Institute of Green Bio Science and Technology, Seoul National University, Pyeongchang, Republic of Korea; 4 Department of Crop Science, Chungnam National University, Yuseong-Gu, Daejeon, Republic of Korea; 5 Kim Jeong Moon Aloe Co. LTD, SeoCho-Gu, Seoul, Republic of Korea; 6 Department of Agricultural Biotechnology, Center for Agricultural Biomaterials, Seoul National University, Seoul, Republic of Korea; 7 Bio-MAX Institute, Seoul National University, Seoul, Republic of Korea; University of Sassari, Italy

## Abstract

*Aloe vera* (Asphodeloideae) is a medicinal plant in which useful secondary metabolites are plentiful. Among the representative secondary metabolites of *Aloe vera* are the anthraquinones including aloe emodin and chrysophanol, which are tricyclic aromatic quinones synthesized via a plant-specific type III polyketide biosynthesis pathway. However, it is not yet clear which cellular responses can induce the pathway, leading to production of tricyclic aromatic quinones. In this study, we examined the effect of endogenous elicitors on the type III polyketide biosynthesis pathway and identified the metabolic changes induced in elicitor-treated *Aloe vera* adventitious roots. Salicylic acid, methyl jasmonate, and ethephon were used to treat *Aloe vera* adventitious roots cultured on MS liquid media with 0.3 mg/L IBA for 35 days. Aloe emodin and chrysophanol were remarkably increased by the SA treatment, more than 10–11 and 5–13 fold as compared with untreated control, respectively. Ultra-performance liquid chromatography-electrospray ionization mass spectrometry analysis identified a total of 37 SA-induced compounds, including aloe emodin and chrysophanol, and 3 of the compounds were tentatively identified as tricyclic aromatic quinones. Transcript accumulation analysis of polyketide synthase genes and gas chromatography mass spectrometry showed that these secondary metabolic changes resulted from increased expression of octaketide synthase genes and decreases in malonyl-CoA, which is the precursor for the tricyclic aromatic quinone biosynthesis pathway. In addition, anti-inflammatory activity was enhanced in extracts of SA-treated adventitious roots. Our results suggest that SA has an important role in activation of the plant specific-type III polyketide biosynthetic pathway, and therefore that the efficacy of *Aloe vera* as medicinal agent can be improved through SA treatment.

## Introduction

**Figure 1:**
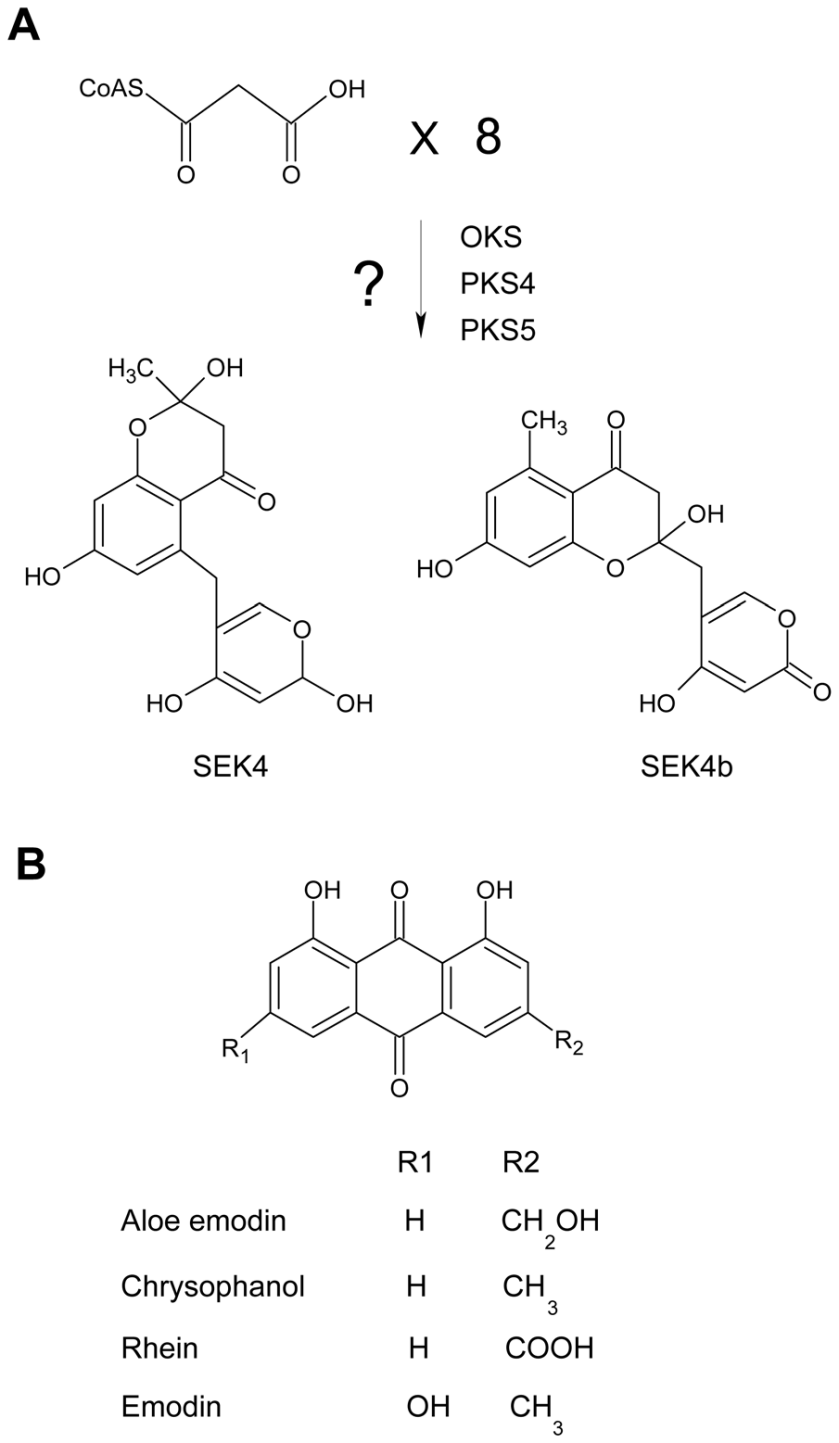
Tentative biosynthesis mechanism for tricyclic aromatic quinones. (A) Production of SEK4 and SEK4b compounds catalyzed by OKS, PKS4, and PKS5 in *E. coli*. (B) The chemical structures of tricyclic aromatic quinone derivatives.

*Aloe vera* (Asphodeloideae) is a medicinal plant in which useful secondary metabolites are abundant [1], [2]. Anthraquinones, which represent one class of *Aloe vera* secondary metabolites, are tricyclic aromatic quinones. Among the naturally occurring anthraquinone derivatives, aloe emodin and chrysophanol are the major compounds [3]. The tricyclic aromatic quinones of aloe have been proposed to be synthesized via the type III polyketide biosynthesis pathway. Recently, novel plant-specific type III polyketide synthases (PKSs), octaketide synthase (OKS), PKS4, and PKS5 were isolated from *Aloe arborescens*, and their functions were examined in *E. coli*. The heterologously expressed enzymes produced SEK and SEK4b, which have an octaketide structure, from eight malonyl-CoAs, but SEK and SEK4b were found to be shunt products of the type II polyketide biosynthesis pathway and have not been detected in plants [4], [5] (Figure 1). This suggested that these novel plant enzymes might potentially be associated with biosynthesis of natural tricyclic aromatic quinones in aloe, but it remains unclear whether these enzymes produce end products such as aloe emodin and chrysophanol *in vivo*.

Identification of secondary metabolites and their derivatives in plants is a first step to determine the associated biosynthetic pathways; however, many remain to be identified because plants are especially rich in secondary metabolites with complex and varied structures [6]. Metabolomics approaches have ushered in a new era for elucidating the complicated secondary metabolism of plants [7], [8], [9], [10]. Among these techniques, mass spectrometry can contribute to deducing the structures of unknown compounds [11], and comprehensive profiling of phenolic compounds including anthraquinones has been carried out in *Cassia* and *Rheum* species using high-performance liquid chromatography-electrospray ionization-mass spectrometry (HPLC-ESI-MS) [12], [13], [14], [15]. In addition, anthraquinone derivatives in rhubarb extract that were biotransformed by rat liver and intestinal bacteria were identified by liquid chromatography-electrospray ionization- tandem mass spectrometry (LC-ESI-MS/MS) [16], [17]. In aloe, metabolite profiling was recently carried out for *Aloe vera* leaves at different developmental stages using gas chromatography-ion trap-mass spectrometry (GC-IT-MS) and ultra-performance liquid chromatography-quadruple time of flight-mass spectrometry (UPLC-QTOF-MS) [18].

In this study, we used GC-MS and UPLC-ESI-MS analysis to investigate the changes in primary and secondary metabolites induced by elicitor treatments of *Aloe vera* adventitious roots. Plant cell culture systems using elicitors have been effective alternatives for production of secondary metabolites, and many abiotic and biotic elicitors have been used to induce or enhance the biosynthesis of secondary metabolites by stimulating plant cellular stress responses [19], [20]. Plant-originated signaling molecules such as salicylic acid (SA), methyl jasmonate (MJ), and ethylene as well as microbe-derived molecules such as polysaccharides, glycoproteins, and inactivated enzymes have been utilized for elicitation of secondary metabolites [21], [22].

Here, we tested the abilities of plant-derived elicitors to activate the type III polyketide biosynthesis pathway in order to improve the production of tricyclic aromatic quinones. We analyzed the changes in metabolic profile and anti-inflammatory activity in the extracts of elicitor-treated adventitious roots. This work shows that SA activates the type III polyketide biosynthesis pathway, resulting in improved production of tricyclic aromatic quinones and increased anti-inflammatory activity. It suggests that the plant specific-type III polyketide biosynthetic pathway is regulated by endogenous SA signaling, and that the efficacy of *Aloe vera* as a medicinal agent can be improved through SA treatment.

## Methods

### Chemicals and reagents

All plant growth media and growth hormones were obtained from Duchefa (Haarlem, The Netherlands). Aloe emodin, chrysophanol, aloin, rhein, and emodin were purchased from Santa Cruz Biotechnology (California, USA). Malonyl-CoA, succinyl-CoA, acetyl-CoA, other chemicals, and solvents were from Sigma-Adrich (St. Louis, USA). All reference standards were dissolved in 100% methanol, and MJ and SA were dissolved in 99.9% (v/v) ethanol. 2-chloroethylphosphonic acid (ethephon) was freshly dissolved in distilled water. Dissolved MJ, SA, and ethephon were sterilized through 0.45 µm membrane filters (Whatman, Tokyo, Japan)

### Optimization of suspension culture conditions and elicitor treatments

Young shoots of *Aloe vera* were provided by Kim Jeong Moon Aloe Co. Ltd (Jeju, Korea). Explants of young leaves were inoculated onto MS medium [23] supplemented with 30 g/L sucrose, 0.5 mg/L 1-naphthaleneacetic acid (NAA), and 0.7 g/L plant agar to induce adventitious roots as described previously [24]. After 3 weeks of culture, the adventitious roots were transferred into 100 mL Erlenmeyer flasks containing 30 mL MS liquid containing 30 g/L sucrose and 0.3 mg/L indole-3-butyric acid (IBA). The pH of medium was adjusted to 5.8 and then autoclaved at 121°C for 15 min. The induced adventitious roots were incubated on an orbital shaker (50 rpm) at 25°C under constant light conditions (light intensity: 7 µE/m^2^s). Various concentrations of MJ, SA, and ethephon were added to the medium to treat 35-d-old adventitious roots.

### Estimate of adventitious root growth

The adventitious roots were harvested every 7 d up to 42 d during growth. Fresh weight was determined to 0.05 g accuracy, and then dry weight (DW) was measured after lyophilization. The growth ratio was calculated as follows: DW of harvested roots - DW of inoculated adventitious roots (initial DW)/initial DW as previously reported [25].

### Quantification of aloe emodin and chrysophanol in intracellular and extracellular

The extraction of aloe emodin and chrysophanol from the culture medium was performed as previously reported with some modifications [26]. Harvested growth medium (30 mL) was supplemented with XAD-4 (0.07 g) and continuously agitated at 125 rpm for 5 days at 25°C. Then, the XAD-4 was collected by vacuum filtration and resuspended in 5 mL 100% ethanol followed by re-incubation for 5 days at 25°C with 125 rpm agitation. The extract in ethanol was concentrated to 500 µL. The HPLC analysis conditions for aloe emodin and chrysophanol within the adventitious roots and culture media were described previously [24]. All samples obtained from the same condition were analyzed with three independent replicates and all experiments were repeated twice.

### Sample preparation for UPLC-ESI-MS

For analysis of secondary and primary metabolites in cultured adventitious roots, lyophilized *Aloe vera* adventitious roots that were treated with 0, 500, 1000, and 2000 µM SA for 24 h (180 mg, n = 5) were extracted with 70% ethanol under sonication for 30 min at room temperature. The extracts were centrifuged at 14000×g for 10 min at room temperature and then completely dried under nitrogen gas. The dried residue was resuspended in 200 µL 70% methanol and filtered through a 0.2 µm polytetrafluoroethylene membrane filter (Whatman, Tokyo, Japan). Each sample was collected in quintuplicate from independent culture flasks and experiments were performed twice.

### Sample preparation for GC-MS

The 50 µL ethanolic extracts were completely dried under nitrogen gas, and agitated with N-methyl-N-(trimethylsilyl) trifluoroacetamide +1% trimethylchlorosilane and pyridine (1∶2) at 60°C for 15 min. The solutions were then transferred into 2 mL glass vials coupled with micro-inserts (Agilent, Santa Clara, CA) and capped immediately.

### Conditions for UPLC-ESI-MS

The extracts were analyzed with a Waters Alliance LC coupled to Quattro microMS on AQUITY UPLC BEH C_18_ columns (2.1×100 mm, 1.7 µm). The UPLC-ESI-MS was carried out using mixtures of 0.1% aqueous formic acid (solvent A) and 100% acetonitrile (solvent B) at 0.1 mL/min flow rate and maintained at 40°C. The elution program was as follows: 30% B at 0 min, 30% B at 5 min, 35% B at 10 min, 70% B at 35 min, 70% B at 45 min, and 100% B at 50 min. The injection volume was 10 µL and capillary voltages were adjusted to +4.0 kV for positive mode and to −4.0 kV for negative mode. MS/MS analysis was performed using collision energy from 5 to 60 eV in positive and negative modes.

### Conditions for GC-MS

The primary metabolites in aloe extracts were analyzed using a 6890 gas chromatograph (Agilent Technologies, CA, USA) coupled with a JMS-GC mate (Jeol, Tokyo, Japan). A DB-5 column (30 m×0.25 mm I.D., 0.25 µm film thickness, HP) was used with helium (99.9999% He) as a carrier gas at a constant flow of 1 mL/min. The oven temperature was held at 60°C for 5 min, ramped to 320°C at a rate of 10°C/min and held for 10 min. One microliter sample was injected in split mode (10∶1). The ionization energy was 70 eV in electron impact mode. The transfer line and ion source temperatures were set at 300°C and 300°C, respectively. After a 300 sec solvent delay, mass spectra were obtained at 20 scans per second with a mass range of 55–600 m/z.

### Data processing and multivariate analysis

All raw data obtained from GC-MS were converted to ASCII format. The raw data were reduced into 6 sec buckets and normalized by the total sum of intensities as previously reported [27]. Raw data files obtained from UPLC-ESI-MS were exported to MZ-mine software version 2.1 and filtered through the Savitzky-Golay filter method to remove the noise. The baseline was then corrected and peaks were detected. Drifted retention time (RT) between replicated samples was adjusted through peak alignment. Finally RT was normalized to reduce the deviation of RT between peak lists, and data alignment was performed using RANSAC aligner.

The peak lists resulting from GC-MS and UPLC-ESI-MS were evaluated using multivariate analysis with SIMCA-P 12.0 (Umetrics, Umeå, Sweden). Unsupervised principal component analysis (PCA) was performed, and supervised partial least square discriminant analysis (PLS-DA) and orthogonal partial least squares-discriminant analysis (OPLS-DA) were processed to compare each elicitor-treated condition and obtain differential metabolites from the elicitor conditions. The major metabolites that were differential between the elicitor conditions were regarded as variable importance in the project (VIP) list and m/z having a cutoff score above the 1 was selected in the PLS-DA model.

Statistical analysis of raw files obtained from GC-MS, UPLC-ESI-MS, and results of other experiments were carried out using Statistica Version 10 (StatSoft Inc., OK, USA), and the Tukey Honestly Significant Difference and Least Significant Difference test were performed at probability level of 0.05.

### Metabolite identification

The compounds were identified based on mass spectra and RT of authentic compounds. Peaks obtained from GC-MS were identified by comparison with spectra of the National Institute of Standards and Technology library on the basis of MS spectra. Among the m/z from UPLC-ESI/MS, major compounds obtained from multivariate or statistical analysis were tentatively identified by MS/MS spectra.

### Isolation and RNA expression analysis of *OKS* genes in *Aloe vera*

Total RNA was extracted by a modified lithium chloride method according to previous reports [28]. The cDNA synthesis was conducted using the Maxime™ RT PreMix (Oligo (dT)_15_ Kit (iNtRON, Sungnam, Korea) according to the manufacturer's protocol. Synthesized cDNA was diluted 1/5 and used as template to isolate *OKS* genes and for real-time PCR.

Full length *OKS* genes were isolated using primers designed from *AaOKS* (Accession number: AY567707) and *AaPKS4* (Accession number: FJ536166.1). PCR was performed as follows: 94°C for 5 min and 35 cycles of 95°C for 30 s, 45°C for 30 s, and 72°C for 1 min 30 s. The obtained PCR products were cloned into T-blunt cloning vector (Solgent, Daejeon, Korea) and sequenced using an ABI 3730 XL DNA Analyzer (Applied Biosystems, CA, USA) with M13 Forward and reverse primers. The inserts of positive clones were amplified to obtain *OKS* candidate genes.

To carry out real time PCR, the *Ubiquitin* gene isolated from *Aloe vera* was used to normalize the C_T_ values of target genes (Accession No. EF539181). Gene-specific primers were designed using the Primer 3 program [29] and specificity was confirmed by sequencing with an ABI 3730 XL DNA Analyzer. PCR reactions were carried out using a Light cycler 480 (Roche, Mannheim, Germany). The thermal cycling conditions were as follows: 95°C for 5 min and 40 cycles of 95°C for 15 s, 58°C for 10 s, and 72°C for 10 s. The primer sequences used to amplify each gene are summarized in Table S1.

Gene expression levels were analyzed by reverse transcription quantitative PCR (RT-qPCR) using gene specific primer sets. Quantities of total RNA were normalized by comparison of band intensities for *Ubiquitin*. Thermal cycling conditions for RT-qPCR were as follows: 94°C for 5 min and 28 cycles of 94°C for 30 s, 58°C for 30 s, and 72°C for 5 min. The amplified PCR products were separated on 2% agarose gel.

### Luciferase activity driven by *COX-2*, *AP-1* and *NF-κB*

The JB6 P+ mouse epidermal cell line which was kindly provided by Dr. Zigang Dong (University of Minnesota, Austin MN) [30] was cultured in monolayers on minimal essential medium (MEM) supplemented with 5% (v/v) fetal bovine serum (FBS) and 0.1% penicillin/streptomycin at 37°C in a humidified atmosphere of 5% CO_2_. The JB6 P+ cells were stably transfected with COX-2, AP-1, or NF-*κ*B luciferase reporter plasmid containing the G418 resistance gene and maintained in 5% FBS-MEM supplemented with 200 mg/mL G418. The cells were seed to 96-well plate, and the plates were incubated in a 5% CO_2_ incubator at 37°C. When cultured cells reached approximately 80% to 90%confluency, the cells were starved with 0.1% FBS-MEM for 24 h. After that, cells were treated with various concentrations (0, 10, 20, 40, and 100 µg/mL) of *Aloe vera* adventitious root extracts (non-treated, 500 µM SA-treated, 1000 µM SA-treated, and 2000 µM SA-treated) for 1 h followed by exposure to UVB (0.05 J/cm^2^) and incubation for 4 h. JB6 P+ cells treated with UVB were disrupted with 100 µL lysis buffer [0.1 M potassium phosphate buffer (pH 7.8), 1% Triton X-100, 1 mM DTT, and 2 mM EDTA]. The luciferase activity was measured using a luminometer (Luminoskan Ascent; Thermo Electron, Helsinki, Finland).

## Results

### Optimization of suspension culture conditions and elicitor effects on accumulation of aloe emodin and chrysophanol in *Aloe vera* adventitious roots

**Figure 2:**
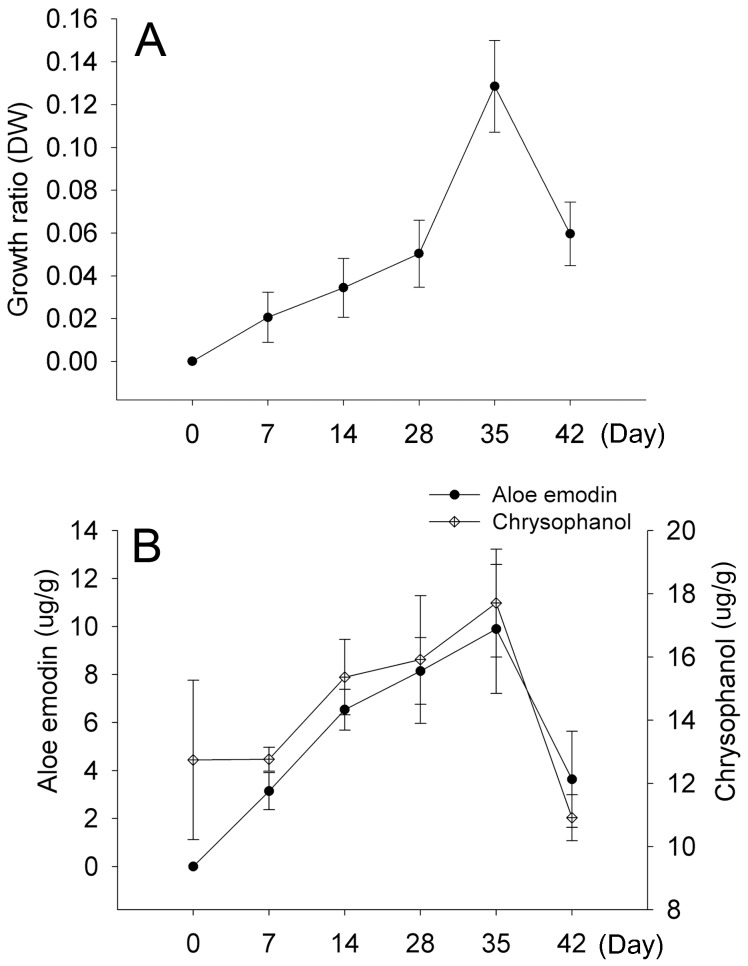
Kinetic analysis of *Aloe vera* suspension culture. (A) Biomass accumulation of *Aloe vera* adventitious roots cultivated in MS liquid medium supplemented with 0.3 mg/L IBA. (B) Accumulation patterns of aloe emodin and chrysophanol in *Aloe vera* adventitious roots cultured on MS medium including 0.3 mg/L IBA. Each value is the mean of replicates and error bars indicate standard deviation.

We began by optimizing the suspension culture conditions for *Aloe vera* adventitious roots prior to elicitation. Three-week-old adventitious roots cultured from solid media were transferred into MS liquid media including 30 g/L sucrose, together with 0.5 mg/L IBA, indole-3-acetic acid, or NAA. After 4 weeks of culture, the adventitious roots were grew normally in MS media supplemented with IAA and IBA, but not in that supplemented with NAA (data not shown). We then tested the influence of IAA or IBA at concentrations of 0, 0.1, 0.3, 0.5, and 1.0 mg/L on adventitious roots, followed by examination of MS, 1/2 MS, 2MS, B5 [31], and SH [32] media (Table S2). After 35 d of cultivation, the maximum biomass of adventitious roots and maximum production of aloe emodin and chrysophanol, as representative secondary metabolites in *Aloe vera*, were found in MS media supplemented with 0.3 mg/L IBA (Figure 2 and Table S2).

**Figure 3:**
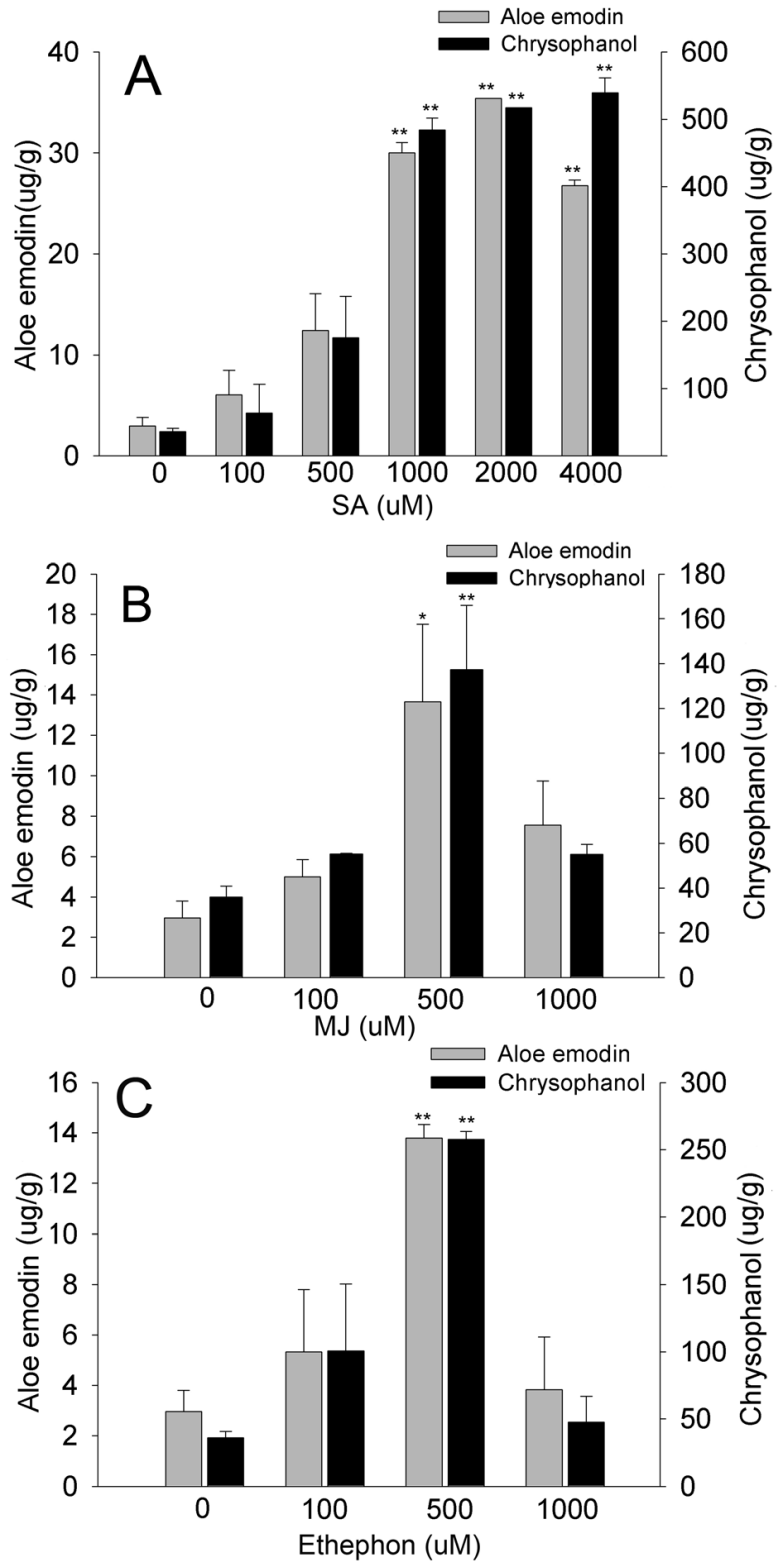
Effect of elicitor treatments on aloe emodin and chrysophanol production in *Aloe vera* adventitious roots. Aloe emodin and chrysophanol production in *Aloe vera* adventitious roots treated with SA (A), MJ (B), and ethephon (C) for 24 h. Data are represented as means of replicate samples ± standard deviation. Statistical analysis was carried out using the Tukey test (* p<0.05, ** p<0.01). Asterisks indicate significant differences compared to aloe emodin and chrysophanol contents obtained from non-treated adventitious roots.

**Figure 4:**
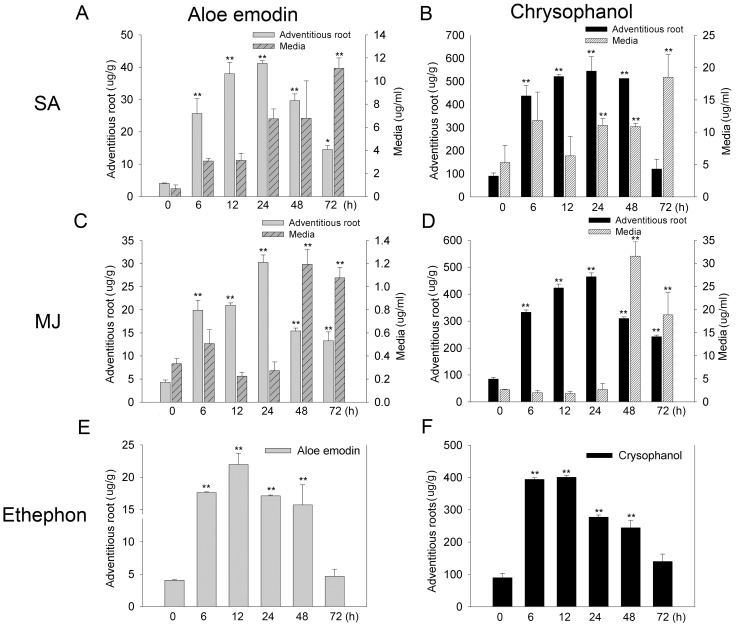
Time-course analysis of aloe emodin and chrysophanol production following elicitation. Content of aloe emodin (A, C, and E) and chrysophanol (B, D, and F) in adventitious roots and culture medium following non and elicitation with 2000 µM SA (A and B), 500 µM MJ (C and D), or 500 µM ethephon (E and F). Data are represented as means of replicate samples ± standard deviation. Statistical analysis was carried out using the Tukey test (* p<0.05, ** p<0.01). Asterisks indicate significant differences compared to aloe emodin and chrysophanol contents obtained from adventitious roots before elicitation.

Next, elicitation conditions were investigated using *Aloe vera* adventitious roots cultured under optimized suspension culture conditions. MJ, SA, and ethaphon were added in various concentrations to 35-d-old adventitious roots, and contents of aloe emodin and chrysophanol in the adventitious roots and in the media were measured, respectively. Maximum production of aloe emodin and chrysophanol occurred in adventitious roots treated with 1000–2000 µM SA (Figure 3). Time course analysis revealed that when adventitious roots were treated with various concentrations of SA, MJ, and ethephon, accumulation of aloe emodin and chrysophanol in adventitious roots was increased by more than 10–11 and 5–13 fold at 24 h, respectively, and that in the growth medium rose at 24–72 h of 2000 µM SA treatment (Figure 4A and 4B). Treatment with 500 µM MJ and 500 µM ethephon also led to increases in aloe emodin and chrysophanol at 24 h (Figure 3B and 3C). With 500 µM MJ, endogenous levels of aloe emodin and chrysophanol steadily increased and spiked at 24 h with over 4–7 fold and 3–5 fold increases, respectively. Exogenous levels of these metabolites increased at 48–72 h following MJ treatment (Figure 4C and 4D). Treatment with 500 µM ethephon induced endogenous aloe emodin and chrysophanol at 12 h, with 5- and 4-fold increases, whereas it did not promote secretion of the these metabolites into the growth culture medium (Figure 4E and 4F). SA treatment showed the most marked effect on production of aloe emodin and chrysophanol, and thus we investigated the responses of the tricyclic aromatic quinone biosynthesis pathway to SA elicitation.

### Primary metabolite analysis using GC-MS and LC-MS

**Figure 5:**
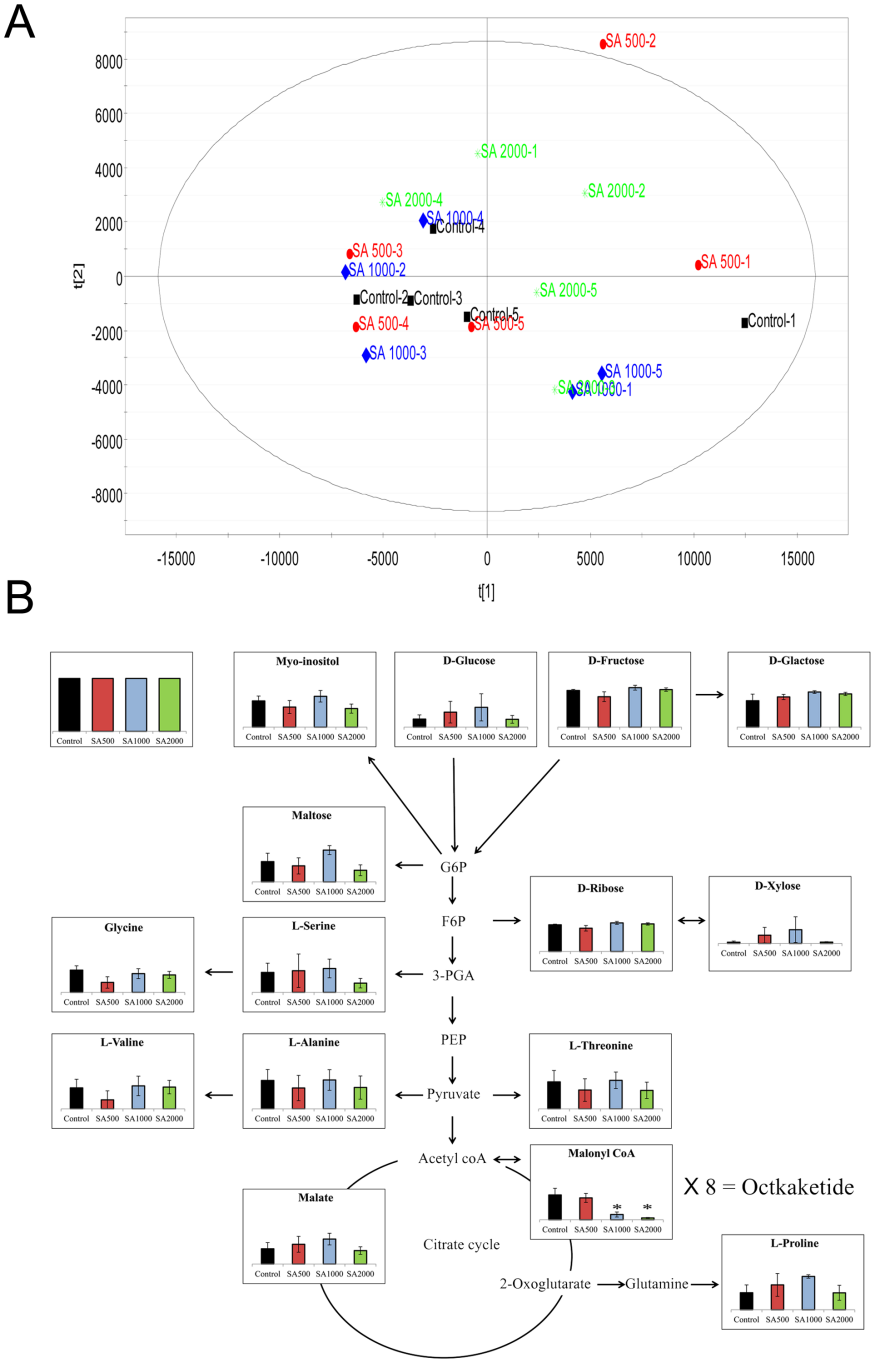
Alteration of primary metabolites in *Aloe vera* adventitious roots in response to SA. (A) OPLS-DA of primary metabolites obtained from adventitious roots treated with 0, 500, 1000, or 2000 µM SA analyzed by GC-MS. (B) Alteration of primary metabolites associated with TCA and glycolysis in response to SA elicitation. The level of malonyl-CoA decreased in a SA dose-dependent manner. Levels of other metabolites did not change in response to SA elicitation. G6P: Glucose-6-phosphate, F6P: Fructose 6-phosphate, PEP: phosphoenolpyruvate.

We speculated that SA might affect primary metabolites that play roles as intermediates in the biosynthetic pathways for secondary metabolites as well as induce considerable changes in secondary metabolites including aloe emodin and chrysophanol. To monitor alterations in primary metabolites including malonyl-CoA, which is a precursor for the biosynthesis of tricyclic aromatic quinones, extracts from *Aloe vera* adventitious roots treated with 0, 500, 1000, and 2000 µM SA for 24 h were analyzed by GC-MS and LC-MS. Primary metabolites profiling analyzed by GC-MS did not reveal any difference upon treatment with different concentration of treated SA. The score plot of OPLS-DA showed that the SA-treatment replicates did not form separate clusters based on peak intensity (Figure 5A). Similar patterns were observed in the PCA and PLS-DA score plots (data not shown). Peaks associated with glycolysis and the tricarboxylic acid cycle (TCA), which is the principal pathway to form malonyl-CoA, were not significantly changed (Figure 5B and Table S3) and were not segregated clearly in the same multivariate analyses (data not shown). Interestingly, LC-MS analysis revealed that malonyl-CoA was remarkably decreased in SA-treated adventitious roots in a concentration-dependent manner, suggesting that consumption of malonyl-CoA is affected by biosynthesis of tricyclic aromatic quinones including aloe emodin and chrysophanol (Figure 5B and Table S2). Succinyl-CoA and acetyl-CoA, which are involved in the TCA cycle, could not be detected in either non-elicited or elicited samples.

### Isolation and gene expression of *Aloe vera OKS* genes

The decrease in malonyl-CoA might result from activation of the type III polyketide biosynthesis pathway. To investigate changes in expression levels of the *OKS*s following SA treatment, the full length cDNAs for *Aloe vera OKS* (*AvOKS*) and *OKS like-1* (*AvOKSL-1*) were isolated from adventitious roots based on the sequences of *Aloe arborescense OKS* (*AaOKS*, Accession number: AY567707) and *PKS4* (*AaPKS4*, Accession number: FJ536166.1). Each *AvOKS* candidate gene had a 1212-bp open reading frame. Compared with *AaOKS*, *AaPKS4*, and *AaPKS5* in *Aloe arborescense*, the deduced amino acid sequences of *AvOKS* and *AvOKSL-1* were 90–99% identical and included conserved active sites such as the chalcone synthase (CHS) active sites (Met 147, Gly 221, Gly 226, and Pro 388), the catalytic triad of CHS (Cys 174, His 316, and Asn 349), gatekeepers (Phe 225 and Phe 275), and Gly 207, Leu 266, and Val 351 (Figure S1 and Table S4) [5].

**Figure 6:**
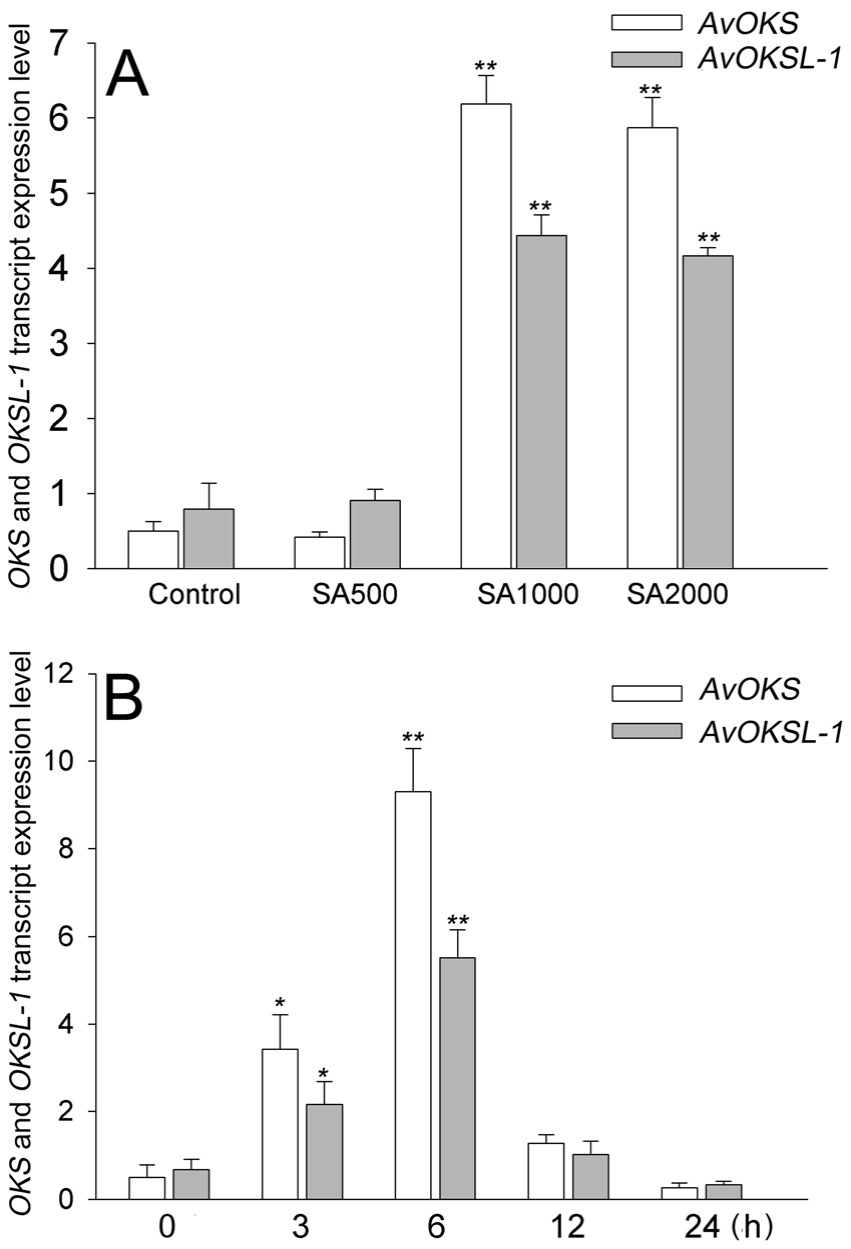
Effect of SA elicitation on transcript accumulation of *OKS* and *OKSL-1*. (A) Transcript accumulation of *OKS* and *OKSL-1* at 6 h of 0, 500, 1000, and 2000 µM SA treatment relative to that of *Ubiquitin*. (B) Time course analysis of gene expression of *OKS* and *OKSL-1* in the presence of 1000 µM SA relative to that of *Ubiquitin*. Each value is the mean of replicates and error bars mean standard deviation. Statistical analysis was carried out using the Tukey test (* p<0.05, ** p<0.01). Asterisks indicate significant differences compared to control groups.

Real-time PCR and RT-qPCR analysis showed that the expression of *AvOKS* and *AvOKSL-1* was increased in proportion to the concentration of treated SA (Figure 6 and Figure S2). The transcripts for *AvOKS* and *AvOKSL-1* were up-regulated more than 6-fold at 6 h of 1000 µM SA elicitation. This suggests that SA induced the expression of *AvOKS* and *AvOKSL-1*, and in turn, the elevated enzyme activity accelerated the condensation of malonyl-CoA, resulting in increased production of tricyclic aromatic quinone derivatives including aloe emodin and chrysophanol.

### Global metabolite analysis

**Figure 7:**
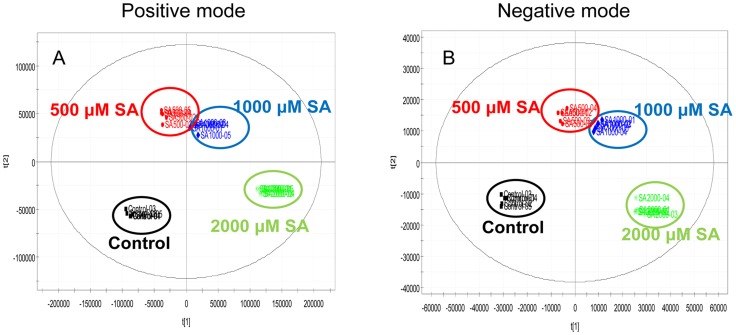
OPLS-DA score plot. OPLS-DA score plots of control (black), 500 µM SA (red), 1000 µM SA (blue), and 2000 µM SA (green)-treated adventitious roots analyzed by UPLC-ESI-MS in positive (A) and in negative (B) mode.

We carried out UPLC-ESI/MS analysis in adventitious roots treated with 0, 500, 1000, or 2000 µM SA for 24 h to identify which metabolites along with aloe emodin and chrysophanol were induced by SA. Based on RT and mass-to-charge ratio (m/z), 1850 and 634 peaks were obtained in positive and negative mode, respectively (Figure S3). According to multivariate results of the UPLC-ESI-MS data sets, the PCA and PLS-DA score plots showed clear segregation between elicitor-treated groups and the untreated group in positive and negative modes (Figure S4 and Table S5). The OPLS-DA analysis also revealed obvious differences among different SA-treated groups in positive and negative modes, indicating that metabolite alterations were influenced by SA concentration (Figure 7 and Table S5). The compounds in the UPLC-ESI-MS data sets induced by SA treatment were classified by one way analysis of variance (p<0.05) in comparison with the control to sort significantly increasing variables. We eliminated variables below 200 m/z because the basic structure of tricyclic aromatic quinones has a molecular weight above 200 m/z. Consequently, we obtained 370 and 130 variables, which were assigned using RT and m/z, in positive and negative modes, respectively (Table S6).

### Identification of SA-induced metabolites using UPLC-ESI-MS/MS

Significantly increased variables in positive and negative modes were analyzed by UPLC-ESI/MS/MS to obtain structural information for unknown compounds induced by SA elicitation. Tentative structures of 37 variables including chrysophanol (SA 18) and aloe emodin (SA 19) were identified in positive and negative modes based on the Chemical Analysis Working Group (Table 1) [33]. These compounds mainly displayed losses of H_2_O (18 Da), CO (28 Da), CH_3_ (15 Da), CH_3_COOH (60 Da), and COOH (45 Da), and in rare cases, degradation of CH_3_CN (41 Da) was detected (Table 1) [16], [34]. Among the identified compounds, variables numbered SA 9, 12, 14, 18, and 28 were designated as VIPs that were capable of discriminating between the samples in PLS-DA models.

**Table 1:** UPLC-ESI/MS/MS data of metabolites induced by SA treatment in *Aloe vera* adventitious roots.

				Fold change^b^				
Mode	No.	Precursor [M+H]^+^	RT^a^	SA500	SA1000	SA2000	MS^2C^	Tentative compounds
negative mode	SA1	299	3.7	38.2^**^	15.4^**^	1.6	MS2-10ev[299]: 299(100), 211(20) MS2-20ev[299]: 240(81)	240 [M-H-CH_3_COOH]^−^
	SA2	287	20.0	4.4*	3.5	3.5	MS2-30ev[287]: 238(100), 209(33), 287(15), 239(9), 269(8)	269 [M-H-H_2_O]^−^ 238 [M-H-H_2_O-CH_2_OH]^−^ 209 [M-H-H_2_O-CH_2_OH-CHO]^−^
	SA3	239	23.2	3.0	4.9	8.9^**^	MS2-30ev[239]: 239(100), 224(15), 210(39), 238(21), 196(16)	224 [M-H-CH_3_]^−^ 210 [M-H-CHO]^−^ 196 [M-H-CH_3_-CO]^−^
	SA4	239	25.2	2.7	4.0	50.0^**^	MS2-30ev[239]: 239(100), 238(44), 224(44)	224 [M-H-CH_3_]^−^
	SA5	239	27.8	5.2	4.4	20.4^**^	MS2-30ev[239]: 238(100), 239(42), 224(34), 210(23)	224 [M-H-CH_3_]^−^ 210 [M-H-CHO]^−^
	SA6	239	31.4	1.4	2.6	26.3^**^	MS2-30ev[239]: 224(100), 196(73), 239(57)	224 [M-H-CH_3_]^−^ 196 [M-H-CH_3_-CO]^−^
	SA7	239	31.9	3.6	4.6	12.9^**^	MS2-30ev[239]: 210(100), 224(16), 238(81), 196(50), 239(19)	224 [M-H-CH_3_]^−^ 210 [M-H-CO]^−^ 196 [M-H-CH_3_-CO]^−^
	SA8	257	10.6	2.6	3.2	5.0*	MS2-30ev[257]: 187(100), 238(73), 215(67), 257(49), 172(35), 239(29)	239 [M-H-H_2_O]^−^ 215 [M-H-C_2_H_2_O]^−^ 187 [M-H-C_2_H_2_O-CO]^−^
	SA9	257	21.1	2.7^**^	2.8^**^	3.8^**^	MS2-30ev[257]: 239(100), 211(36), 257(23), 224(21), 172(22)	239 [M-H-H_2_O]^−^ 224 [M-H-H_2_O-CH_3_]^−^ 211 [M-H-H_2_O-CO]^−^
	SA10	269	9.7	1.3	1.2	4.0*	MS2-30ev[269]: 241(100), 195(61), 269(43), 213(46)	241 [M-H-CO]^−^ 213 [M-H-2CO]^−^ 195 [M-H-2CO-H_2_O]^−^
	SA11	269	21.3	7.7^**^	7.8^**^	11.1^**^	MS2-30ev[269]: 197(100), 241(58), 225(44), 269(43)	241 [M-H-CO]^−^ 225 [M-H-CO_2_]^−^ 197 [M-H-2CO]^−^
	SA12	273	9.4	6.5^**^	7.1^**^	7.8^**^	MS2-20ev[273]: 255(100), 237(56), 273(36), 226(35)	255 [M-H-H_2_O]^−^ 237 [M-H-2H_2_O]^−^ 226 [M-H-H_2_O-CHO]^−^
	SA13	301	4.8	1.4	2	5.5^**^	MS2-25ev[301]: 257(100), 215(70), 239(54), 187(28), 301(17)	257 [M-H-COO]^-^ 239 [M-H-COO-H_2_O]^−^
	SA14	311	29.2	1.5	1.6*	2.0^**^	MS2-35ev[311]: 224(100), 253(20), 225(15), 311(2)	253 [M-H-CH_3_COCH_3_]^−^ 224 [M-H-CH_3_COCH_3_-CHO]^−^
	SA15	313	8.7	8.9*	5.3	11.0^**^	MS2-30ev[313]: 269(100), 313(25)	269 [M-H-COOH]^−^ 241 [M-H-COOH-CO]^−^ 225 [M-H-COOH-CO-O]^−^ 197 [M-H-COOH-2CO-O]^−^
							MS2-30ev[269]: 225(100), 241(75), 197(59), 269(25)	
	SA16	327	22.8	3.2^**^	2.9^**^	4.5^**^	MS2-35ev[327]: 240(100), 239(86), 211(58), 269(19)	269 [M-H-CH_3_COCH_3_]^−^ 240 [M-H-CH_3_COCH_3_-CHO]^−^
	SA17	329	16.3	9.7	18.4	24.2^**^	MS2-20ev[329]: 283(100), 268(36), 240(20)	283 [M-H-COOH_2_]^−^ 268 [M-H-COOH_2_-CH_3_]^−^ 240 [M-H-COOH_2_-CH_3_-CO]^−^
	SA18^d^	253	36.6	3.1	5.6^**^	6.9^**^	MS2-25ev[253]: 225(100), 253(11)	255 [M-H-CO]^−^
	SA19^e^	269	19.6	3.8^**^	5.2^**^	4.9^**^	MS2-22ev[269]: 269(100), 240(92)	240 [M-H-CHO]^−^
Positive mode	SA20	259	8.1	2.2*	2.0	1.6	MS2-20ev[259]: 241(100)	241 [M+H-H_2_O]^+^ 213 [M+H-CO]^+^ 185 [M+H-2CO]^+^
							MS2-20ev[241]: 213(100), 241(54), 185(26)	
	SA21	275	8.5	40.8^**^	19.2	1.2	MS2-20ev[275]: 227(100), 275(12), 255(2)	255 [M+H-H_2_O-H_2_]^+^ 227 [M+H-H_2_O-H_2_-CO]^+^
	SA22	257	20.1	8.2*	7.1	6.8	MS2-20ev[257]: 211(100), 239(51), 257(22)	239 [M+H-H_2_O]^+^ 211 [M+H-CO]^+^ 185 [M+H-2CO]^+^
							MS2-20ev[239]: 211(100), 239(24), 185(27)	
	SA23	358	39.6	1.9^**^	1.4	0.5	MS2-10ev[358]: 317(100)	358 [M+H-CH_3_CN]^+^ 273 [M+H-CH_3_CN-COO^−^]^+^ 258 [M+H-CH_3_CN-COO^−^-CH_3_]^+^
							MS2-30ev[317]: 317(100), 235(85), 273(71), 258(19)	
	SA24	255	29.4	3.7	6.4*	5.8*	MS2-30ev[255]: 181(100), 227(40), 209(20), 255(7)	227 [M+H-CO]^+^ 209 [M+H-CO-H_2_O]^+^ 181 [M+H-2CO-H_2_O]+
	SA25	240	30.6	3.2	3.7*	3.0*	MS2-20ev[240]: 240(100), 194(18), 222(9), 212(2)	222 [M+H-H_2_O]^+^ 212 [M+H-CO]^+^ 194 [M+H-CO-H_2_O]^+^
	SA26	240	38.5	3.1	4.6^**^	4.2^**^	MS2-30ev[240]: 194(57), 240(10), 222(4), 212(3)	
	SA27	256	38.5	3.2	5.0^**^	4.6^**^	MS2-30ev[256]: 181(100), 209(17), 227(8), 256(7)	227 [M+H-CHO]^+^ 209 [M+H-CHO-H_2_O]^+^ 181 [M+H-CHO-H_2_O-CO]^+^
	SA28	443	6.9	2.1	2.5	9.7^**^	MS2-20ev[433]: 443(100), 233(28), 353(18), 413(16)	413 [M+H-CH_2_O]^+^ 353 [M+H-CH_2_O-CH_3_COOH]^+^ 233 [M+H-CH_2_O-CH_3_COOH-Glc]^+^
	SA29	351	8.3	2	6.4*	6.4*	MS2-20ev[315]: 297(100)	297 [M+H-H_2_O]^+^ 269 [M+H-H_2_O-CO]^+^ 241 [M+H-H_2_O-2CO]^+^ 213 [M+H-H_2_O-3CO]^+^
							MS2-30ev[297]: 213(100), 241(66), 185(38), 269(14), 297(5)	
	SA30	257	9.3	9.9*	11.6^**^	15.3^**^	MS2-20ev[257]: 229(100), 239(21), 257(12)	239 [M+H-H_2_O]^+^ 229 [M+H-CO]^+^ 211 [M+H-CO-H_2_O]^+^ 201 [M+H-2CO]^+^
							MS2-20ev[229]: 229(100), 211(80), 201(32)	
	SA31	317	11.5	1.3	1.3	2.8*	MS2-20ev[317]: 285(100)	285 [M+H-CH_3_OH]^+^ 267 [M+H-CH_3_OH-H_2_O]^+^ 257 [M+H-CH_3_OH-CO]^+^ 239 [M+H-CH_3_OH-H_2_O-CO]^+^ 229 [M+H-CH_3_OH-2CO]^+^ 211 [M+H-CH_3_OH-2CO-H_2_O]^+^ 183 [M+H-CH_3_OH-3CO-H_2_O]^+^
							MS2-20ev[285]: 183(100), 211(47), 285(35), 239(29), 267(26), 229(5), 257(2)	
	SA32	259	18.8	9.8	11.8	27.0^**^	MS2-20ev[259]: 241(100)	241 [M+H-H_2_O]^+^ 223 [M+H-2H_2_O]^+^ 213 [M+H-H_2_O-CO]^+^ 195 [M+H-2H_2_O-CO]^+^
							MS2-20ev[241]: 241(100), 223(49), 195(44)	
	SA33	259	21.2	4.5	3.8	5.9*	MS2-20ev[259]: 241(100), 223(84), 195(31), 213(9), 259(3)	
	SA34	271	21.4	4.1	4.5	5.5*	MS2-30ev[271]: 173(100), 201(70), 229(50), 271(6)	229 [M+H-C_2_H_2_O]^+^ 201 [M+H-C_2_H_2_O-CO]^+^ 173 [M+H-C_2_H_2_O-2CO]+
	SA35	297	22.9	2.9	2.7	3.2*	MS2-20ev[297]: 297(100), 269(90), 241(53), 213(31), 185(10)	269 [M+H-CO]^+^ 241 [M+H-2CO]^+^ 213 [M+H-3CO]^+^ 185 [M+H-4CO]^+^
	SA36	256	36.7	3.2	2.6	9.5*	MS2-30ev[256]: 228(32), 210(21), 256(11)	228 [M+H-CO]^+^ 210 [M+H-CO-H_2_O]^+^
	SA37	277	40.6	7.3	30.5	63.0^**^	MS2-20ev[277]: 277(56), 249(30), 241(21), 259(16), 226(10),	259 [M+H-H_2_O]^+^ 249 [M+H-CO]^+^ 241 [M+H-2H_2_O]^+^ 226 [M+H-2H_2_O-CH_3_]^+^

^a^ RT, Retention time.

^b^ Average intensity relative to average control intensity.

^C^ Main fragments (Relative intensity).

^d^ SA 18 was identified as chrysophanol confirmed using authentic standard.

^e^ SA 19 was identified as aloe emodin confirmed using authentic standard.

*Asterisk indicates significantly difference relative to control according to Tukey's test (p<0.05 *, p<0.01 **).

Chrysophanol (SA 18) and aloe emodin (SA 19) were observed in adventitious roots treated with 1000∼2000 µM SA. Chrysophanol (SA 18) was detected at 36.4 min, predominately due to the elimination of a CO residue to produce 225 m/z. A MS/MS spectra of deprotonated aloe emodin (SA 19) was monitored at 19.5 min and produced one fragment at 240 m/z by loss of CHO. These MS/MS fragment patterns of aloe emodin and chrysophanol were the same as those of the authentic compounds.

A total of 3 compounds (SA 11, 15, and SA 16) were highly likely to be tricyclic aromatic quinone derivatives. The MS/MS fragment patterns of SA 11 and SA 15 were similar to that of emodin, and SA 16 showed a similar fragment pattern to that of aloe emodin. SA 11 appeared at 21.2 min and gave a deprotonated molecule at 269 m/z. Those MS/MS spectra produced daughter ions of 241 m/z [M-H-CO]^−^, 213 m/z [M-H-2CO]^−^, and 195 m/z [M-H-2CO-H_2_O]^−^. This fragment pattern was similar to that of emodin [13], but the RT was different from that of the pure standard compound, implying that the compound was not emodin per se but might have a similar structure. The SA 15 compound eluted at 8.6 min and produced a deprotonated daughter molecule at 313 m/z. The base peak was further cleaved into 269, 241, 225, and 197 m/z ions. The 269 m/z resulted from elimination of COOH adduct. The 241 m/z ion was produced by disassociation of CO, followed by degradation of a hydroxyl group to form 225 m/z and a CO adduct to give 197 m/z. We tentatively determined that the COOH substitution might exist in a structure resembling emodin [13]. In addition, the spectrum of SA 16 showed a similar pattern to aloe emodin. The initial fragment arose from disassociation of CH_3_COCH_3_ to produce 269 m/z and cleavage of CHO to form 240 m/z as the base peak. The corresponding structure is considered to resemble aloe emodin with a CH_3_COCH_3_ substitution.

### The anti-inflammatory activity of SA-treated *Aloe vera* adventitious roots

Metabolite profiling analysis revealed that a number of metabolites including aloe emodin and chrysophanol were induced by SA elicitation. Tricyclic aromatic quinones such as aloe emodin and chrysophanol have been reported to possess anti-inflammatory activity [35]. Based on these results, we investigated whether SA treatment of adventitious roots led to enhancement of anti-inflammatory activity in UVB-treated mouse skin cells, which are a well-established cell line for screening anti-inflammatory agents.

**Figure 8:**
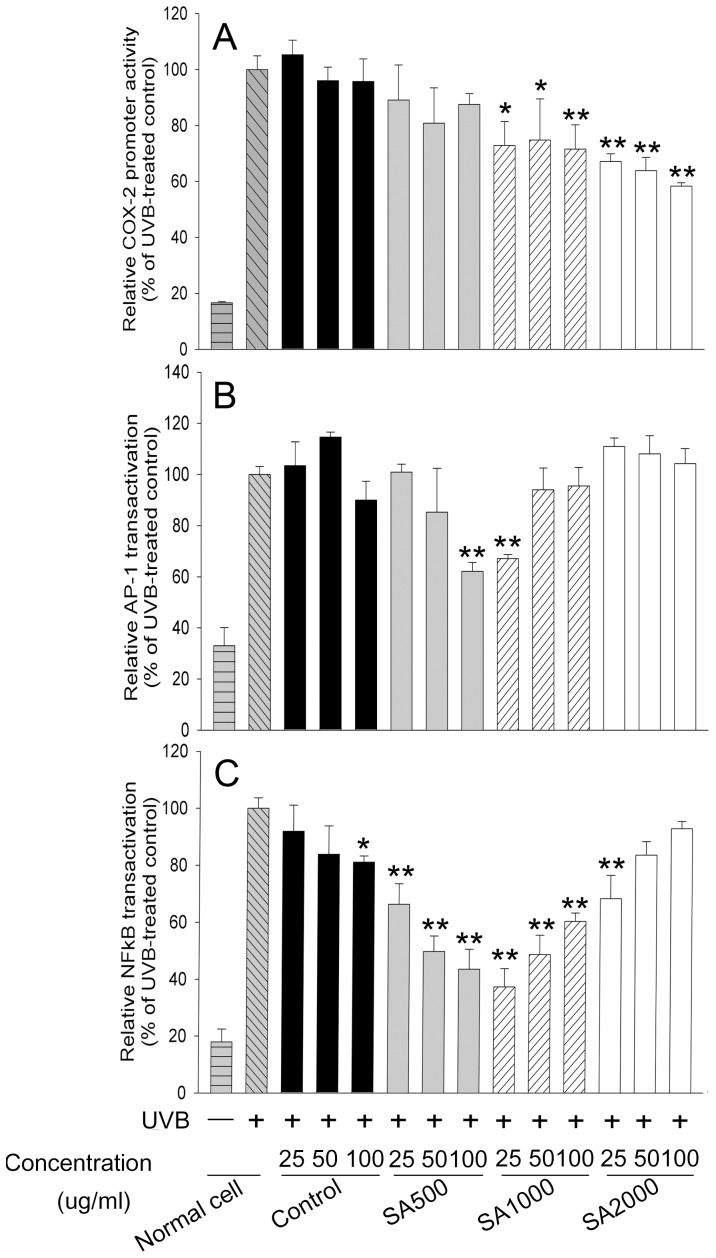
Effect of extracts from SA-treated adventitious roots on UVB-induced expression in mouse skin cells. UVB-exposed JB6 P+ cells that were stably transfected with plasmids containing the luciferase reporter gene fused to the *COX-2* promoter (A), the *AP-1* gene (B), or the *NF- κB* gene (C) were incubated with extract from 0, 500, 1000, or 2000 µM SA-treated adventitious roots for 1 h and harvested after 4 h. Data are represented as means of replicate samples ± standard deviation. Statistical analysis was carried out with the Tukey test (* p<0.05, ** p<0.01). Asterisks indicate significant differences compared to groups treated with UVB alone. Control indicates the extract from SA-untreated adventitious roots.

All extracts obtained from non-treated and SA-treated adventitious roots maintained mouse skin cell viability at 10–100 µg/mL concentrations (Figure S6). Extracts obtained from 1000 µM SA-treated and 2000 µM SA-treated adventitious roots at 25–100 µg/mL suppressed the UVB-induced promoter activity of *COX-2* (Figure 8). UVB-induced transactivation of *NF-κB* and *AP-1* was strongly repressed by extracts from 500 µM SA-treated adventitious roots at 100 µg/mL and 1000 µM SA-treated adventitious roots at 25 µg/mL, respectively (Figure 8). Although *NF-κB* was slightly inhibited by the extract from non-treated adventitious roots at 100 µg/mL, the extract could not suppress promoter activity of *COX-2* and transactivation of *AP-1*. These results indicated that anti-inflammatory activity was enhanced in the adventitious root extracts by SA elicitation, resulting from the induction of a number of metabolites with anti-inflammatory activity. In addition, anti-inflammatory activity was dependent on the concentration of SA used to treat adventitious roots. Although we could identify the metabolites induced by SA treatment as described above, further detailed study is needed to confirm which metabolites account for the anti-inflammatory activity.

## Discussion

In this study, we investigated metabolic changes and enhancement of anti-inflammatory activity in SA-treated *Aloe vera* adventitious roots. Prior to this series of experiments, there was a need for development of *in vitro* cell culture systems for *Aloe vera* in order to retain cell lines in a controlled environment. Although previous studies attempted to optimize callus induction on solid media in *Aloe* species [36], [37], suspension culture had been limited, because phenolic compounds released from the cultured cells eventually led to cell death [38]. In this work, we optimized suspension culture conditions for *Aloe vera* adventitious roots and overcame heavy browning (Figure 2 and Table S2). Using the adventitious roots, we examined the elicitation effects of MJ, SA, and ethephon on the production of aloe emodin and chrysophanol.

MJ, SA, and ethephon are plant-derived elicitors that mediate the signal transduction involved in plant defense responses [39]. SA is associated with pathogen-related defense mechanisms and is required for establishment of plant systemic acquired resistance. Treatment with exogenous SA was previously reported to induce secondary metabolites, e.g. anthraquinones in *Rubia cordifolia* [39], soluble phenolic compounds in *Matricaria chamomilla* and *Salvia miltiorrhiza* [40], [41], podophyllotoxin in *Linum albums* [42] and artemisinin in *Artemisia annua L* [43]. In our study, the production of aloe emodin and chrysophanol was increased by all of these elicitors, but SA remarkably elevated the level of aloe emodin and chrysophanol by more than 10–11 and 5–13 fold at 24 h, implying defense-related function of the quinone compounds (Figure 3 and Figure 4).

We performed UPLC-ESI/MS/MS analysis to identify additional SA-induced tricyclic aromatic quinones along with aloe emodin and chrysophanol. We identified 37 novel compounds induced by SA elicitation through MS/MS fragmentation, and three compounds were determined likely to have tricyclic aromatic quinone structures (Table 1). Our analysis of overall primary metabolite profiles and transcripts of *OKS* genes provided conclusive information about key components in the type III polyketide biosynthesis pathway that was previously uncertain in plants. In previous reports, it was predicted that tricyclic aromatic quinones such as aloe emodin and chrysophanol were synthesized from the precursor of malonyl-CoA in a process mediated by OKSs [4], [5]. However the activity of the plant OKS enzymes was tested in *E. coli*, and it remained unclear whether the condensation process could indeed produce the end products in aloe, as opposed to the shunt products SEK and SEK4b (Figure 1). Our results showed that aloe emodin and chrysophanol biosynthesis correlated with *OKS* gene expression and malonyl-CoA content, supporting the hypothesis that the condensation of malonyl-CoA mediated by OKSs results in production of tricyclic aromatic quinones in *Aloe vera* (Figure 3,4,5,6, and Table S3). On the other hand, some tricyclic aromatic quinones were not induced following SA elicitation. Rhein and emodin were decreased or not changed following SA elicitation, respectively (data not shown), and indicating that SA elicitation might also affect the downstream pathway of the condensation process.

Our metabolite analysis suggests that there is cross-talk in SA and MJ signaling in *Aloe vera*. UPLC-ESI-MS based-analysis showed clear differences between 500 µM SA-treated and 500 µM MJ-treated groups in positive mode (Figure S5 and Table S5). This reveals that SA and MJ can lead to different responses in overall metabolite accumulation in *Aloe vera* adventitious roots. In many cases, SA and MJ have been found to act antagonistically [44], but some cooperative activity was observed in naphthodianthrone and phloroglucinol production in *Hypericum* species and ginsenoside accumulation in *Panax ginseng* [45], [46]. In *Aloe vera*, the biosynthesis of many secondary metabolites was SA- or MJ-dependently regulated, but some metabolites such as tricyclic aromatic quinones seemed to be cooperatively regulated.

Finally, we examined whether SA elicitation enhanced the anti-inflammatory activity of adventitious root extracts. We verified anti-inflammatory effects using JB6 P+ cells and the UVB model, which is suitable for examining chemopreventive effects of phytochemicals [47], [48], [49]. Our results showed that anti-inflammatory activity was affected differently by extracts from adventitious roots treated with different concentrations of SA (Figure 8). This indicates that SA-induced metabolites including aloe emodin and chrysophanol might regulate anti-inflammatory activity in UVB-exposed mice skin cells. Our metabolite profiling data revealed that the identified compounds mainly possessed hydroxy, hydroxymethyl, and carboxyl groups. There is accumulating evidence that the presence of these residues might have an effect on inflammation or angiogenesis associated with inflammation [50], [51], [52]. For example, the presence of 2 hydroxy groups located in aloe emodin might play an important role in its anti-inflammatory activity [51]. Emodin with hydroxyl group at C-6 position, rhein with carboxyl groups at C-6 position, and aloe emodin with hydroxymethyl group at C-3 position might contribute those anti-angiogenetic properties [50], [51]. These types of evidence might partially account for the enhancement of anti-inflammatory activity in SA-treated adventitious roots.

*Aloe vera* is a well-known commercial crop; however, only a few metabolomic approaches have been applied to it. In this study, based on an optimized cell culture system for *Aloe vera*, we showed that SA elicitation led to activation of the tricyclic aromatic quinone biosynthesis pathway and the accumulation of secondary metabolites. Moreover, the extracts from SA-treated *Aloe vera* had enhanced anti-inflammatory activity in UVB-treated mouse skin cells. Taken together, these results provide a possible biological function of quinones related to SA-dependent defense responses and reveal the potential of SA-induced metabolites as chemopreventive agents.

## Supporting Information

Figure S1 **Alignment of amino acid sequences of** ***Aloe vera*** **OKS and OKSL-1.** OKS and OKSL-1 sequences from *Aloe vera* were compared with OKS from *Aloe arborescens* (Accession: AY567707.1), PKS4 from *Aloe arborescens* (Accession: FJ536166.1), and PKS5 from *Aloe arborescens* (Accession: FJ536167.1).
(TIF)

Figure S2 **Expression levels of** ***OKS*** **and** ***OKSL-1*** **in response to SA treatment.** RT-qPCR expression profile of *OKS* and *OKSL-1* at 6 h of 0, 500, 1000, and 2000 µM SA treatment (Lane: Cont, SA500, SA1000, and SA2000) and time course analysis of gene expression of *OKS* and *OKSL-1* in the presence of 1000 µM SA (Lane: 0, 3hr, 6hr, 12hr, and 24hr). Quantities of total RNA were normalized by comparison with the band intensity for *Ubiquitin*, and the PCR products for *Ubiquitin*, *OKS*, and *OKSL-1* were separated on 2% agarose gels.
(TIF)

Figure S3 **Chromatograms obtained from UPLC-ESI-MS in positive and negative modes.**
(TIF)

Figure S4 **PCA and PLS-DA score plots.** The PCA (A and C) and PLS-DA (B and D) score plots of control (black), 500 (red), 1000 (blue), and 2000 (green) µM SA-treated adventitious roots analyzed by UPLC-ESI-MS in positive (A and B) and negative (C and D) mode.
(TIF)

Figure S5 **PCA, PLS-DA, and OPLS-DA score plots**. PCA (A and D), PLS-DA (B and E), and OPLS-DA (C and F) score plots of control (black), 500 µM SA (blue), and 500 µM MJ (red)-treated adventitious roots analyzed by UPLC-ESI-MS in positive (A, B, and C) and negative (D, E, and F) mode.
(TIF)

Figure S6 **Effect of extracts obtained from elicitor-treated adventitious roots on JB6 P+ cell viability.** Extracts were obtained from *Aloe vera* adventitious roots untreated (Control) or treated with 500 µM SA, 1000 µM SA, or 2000 µM SA. JB6 P+ cells were treated with the indicated amounts of each extract for 4 h, and then 20 µL CellTiter 96 Aqueous One solution was added to the cells and they were incubated for an additional 4 h. Cell viability was subsequently measured at 492 and 690 nm. Data are represented as means of replicate samples ± standard deviation. Statistical analysis was carried out using the Tukey test (* p<0.05, ** p<0.01). Asterisks indicate significant differences compared to control groups.
(TIF)

Table S1 **Primer sets used in this study.**
(DOCX)

Table S2 **Effect of plant hormones and media on growth of** ***Aloe vera*** **adventitious roots and accumulation of aloe emodin and chrysophanol after 35 days**.
(DOCX)

Table S3 **Alteration of primary metabolites in** ***Aloe vera*** **adventitious roots following SA treatment.**
(DOCX)

Table S4 **Comparison between amino acid sequences identities of OKS and OKSL-1 from** ***Aloe vera*** **and OKS, PKS4, and PKS5 from** ***Aloe arborescens*** **.**
(DOCX)

Table S5 **Statistical parameters of PCA, PLS-DA, and OPLS-DA in positive and negative modes.**
(DOCX)

Table S6 **Statistically significant peak numbers.**
(DOCX)
