# Supplementary figures and images for: Enhancement of Anti-Inflammatory Activity of Aloe vera Adventitious Root Extracts through the Alteration of Primary and Secondary Metabolites via Salicylic Acid Elicitation
Source: PLoS One. 2013 Dec 16;8(12):e82479. doi: 10.1371/journal.pone.0082479 (PMC3865001; doi:10.1371/journal.pone.0082479)

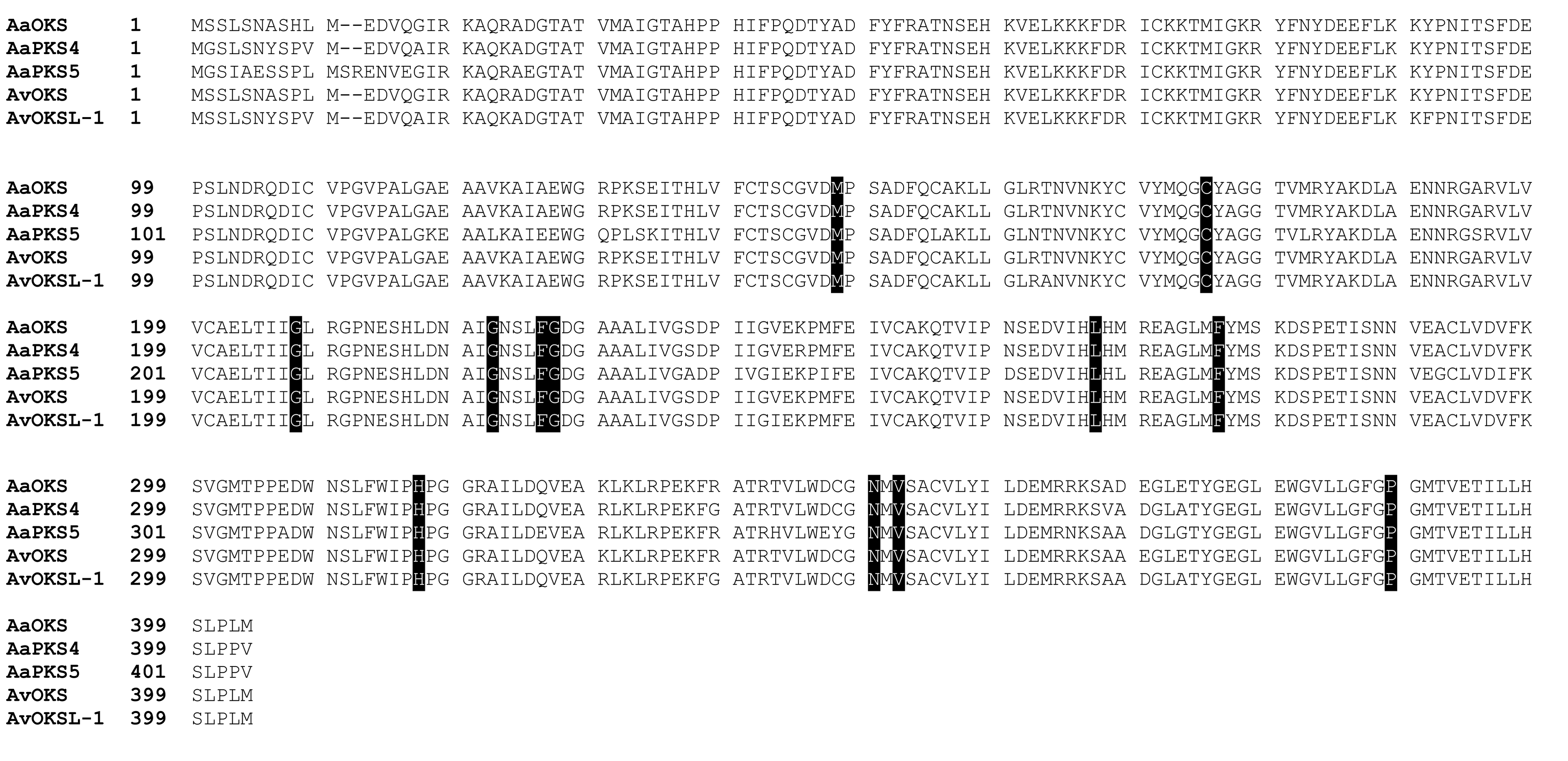

Supplement: Figure S1 — Alignment of amino acid sequences of Aloe vera OKS and OKSL-1. OKS and OKSL-1 sequences from Aloe vera were compared with OKS from Aloe arborescens (Accession: AY567707.1), PKS4 from Aloe arborescens (Accession: FJ536166.1), and PKS5 from Aloe arborescens (Accession: FJ536167.1). (TIF) [file pone.0082479.s001.tif]

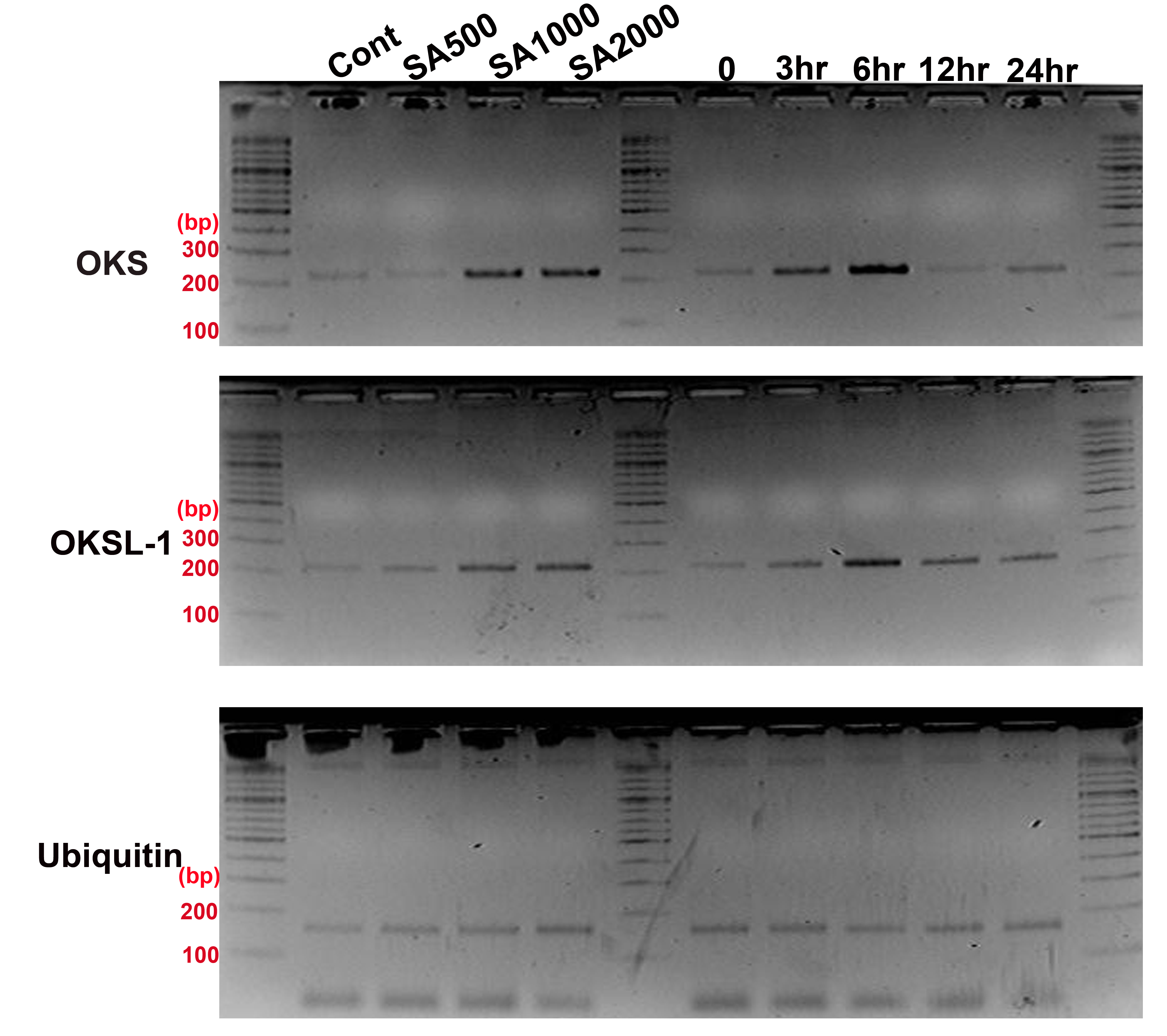

Supplement: Figure S2 — Expression levels of OKS and OKSL-1 in response to SA treatment. RT-qPCR expression profile of OKS and OKSL-1 at 6 h of 0, 500, 1000, and 2000 µM SA treatment (Lane: Cont, SA500, SA1000, and SA2000) and time course analysis of gene expression of OKS and OKSL-1 in the presence of 1000 µM SA (Lane: 0, 3hr, 6hr, 12hr, and 24hr). Quantities of total RNA were normalized by comparison with the band intensity for Ubiquitin, and the PCR products for Ubiquitin, OKS, and OKSL-1 were separated on 2% agarose gels. (TIF) [file pone.0082479.s002.tif]

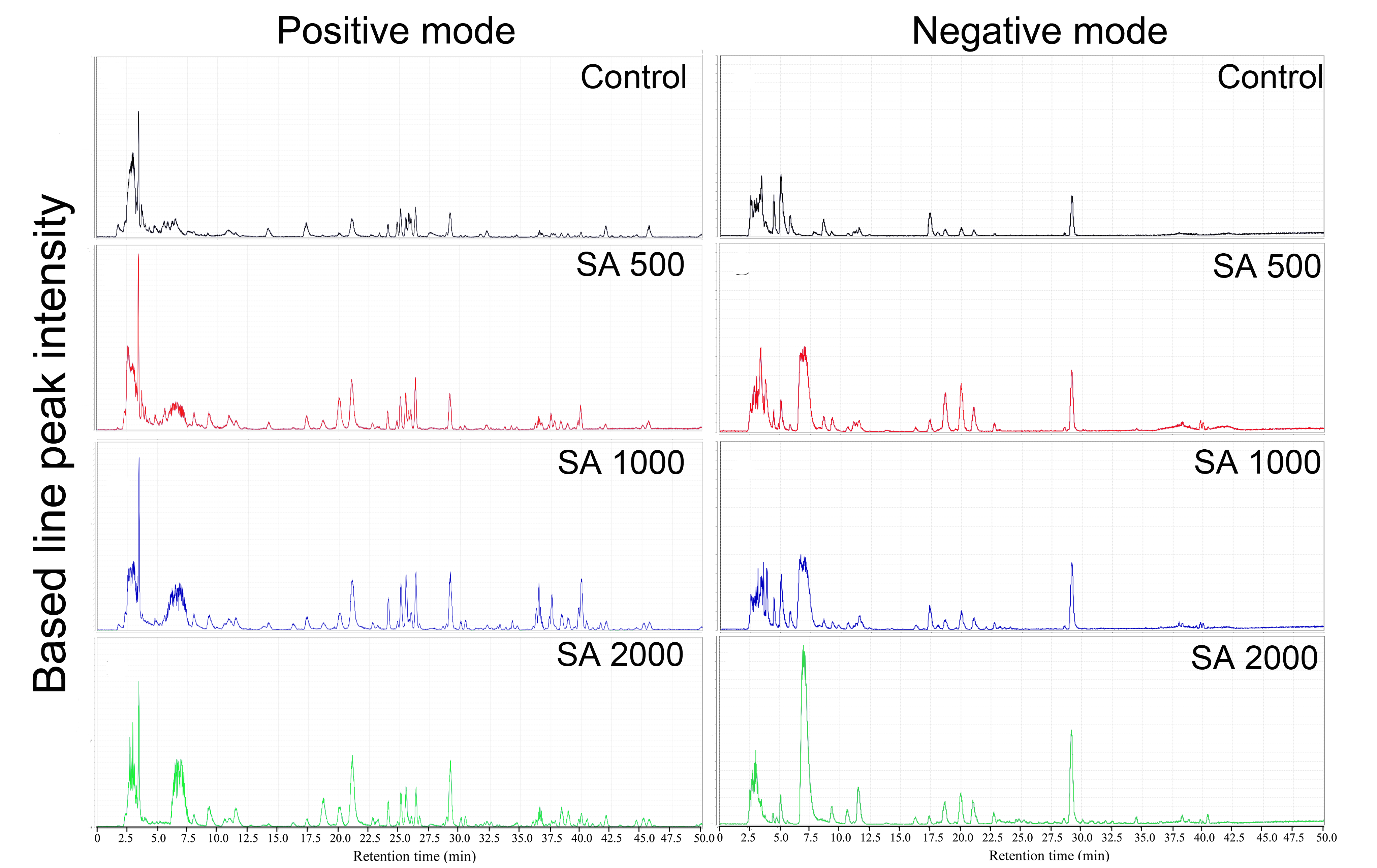

Supplement: Figure S3 — Chromatograms obtained from UPLC-ESI-MS in positive and negative modes. (TIF) [file pone.0082479.s003.tif]

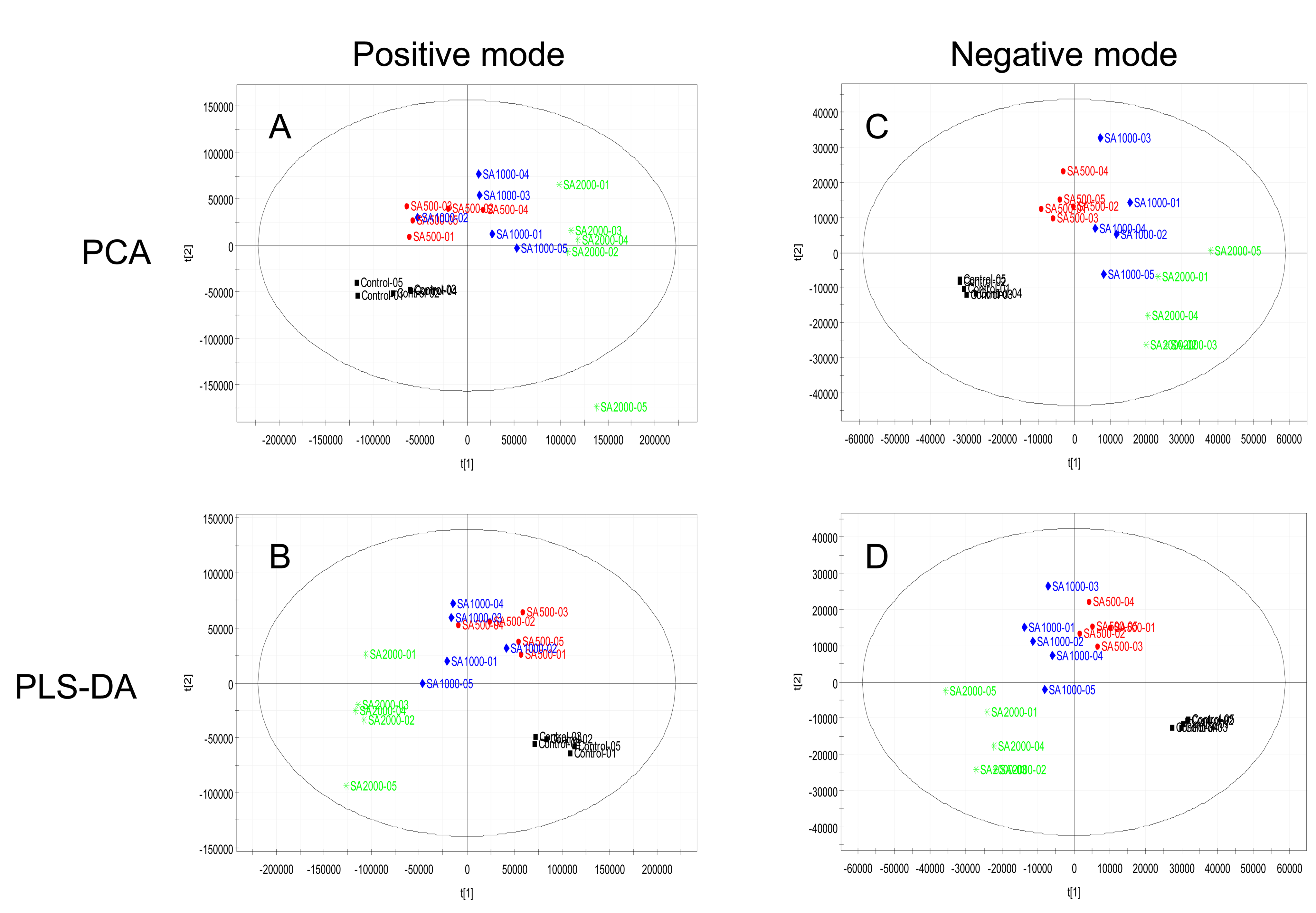

Supplement: Figure S4 — PCA and PLS-DA score plots. The PCA (A and C) and PLS-DA (B and D) score plots of control (black), 500 (red), 1000 (blue), and 2000 (green) µM SA-treated adventitious roots analyzed by UPLC-ESI-MS in positive (A and B) and negative (C and D) mode. (TIF) [file pone.0082479.s004.tif]

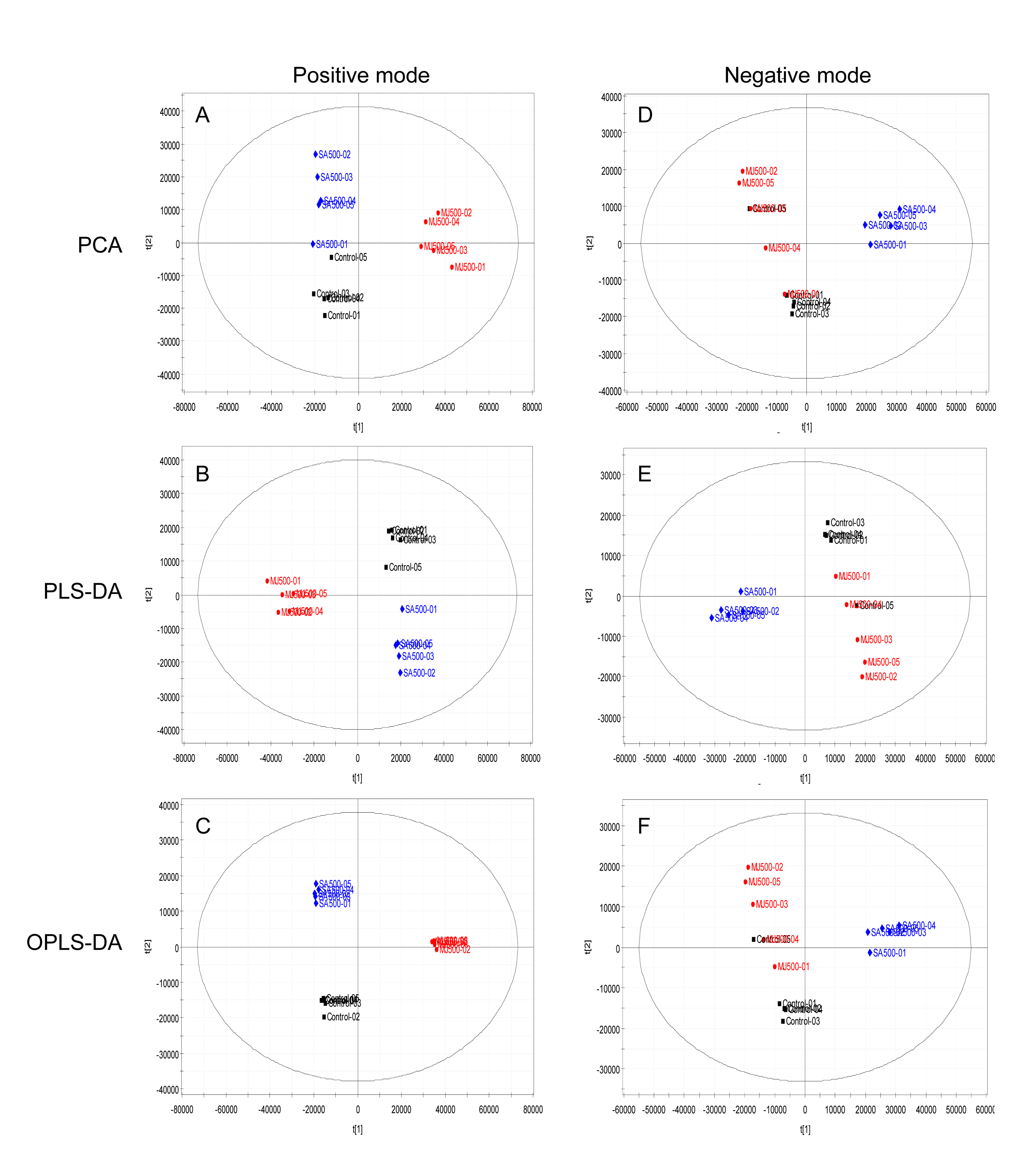

Supplement: Figure S5 — PCA, PLS-DA, and OPLS-DA score plots. PCA (A and D), PLS-DA (B and E), and OPLS-DA (C and F) score plots of control (black), 500 µM SA (blue), and 500 µM MJ (red)-treated adventitious roots analyzed by UPLC-ESI-MS in positive (A, B, and C) and negative (D, E, and F) mode. (TIF) [file pone.0082479.s005.tif]

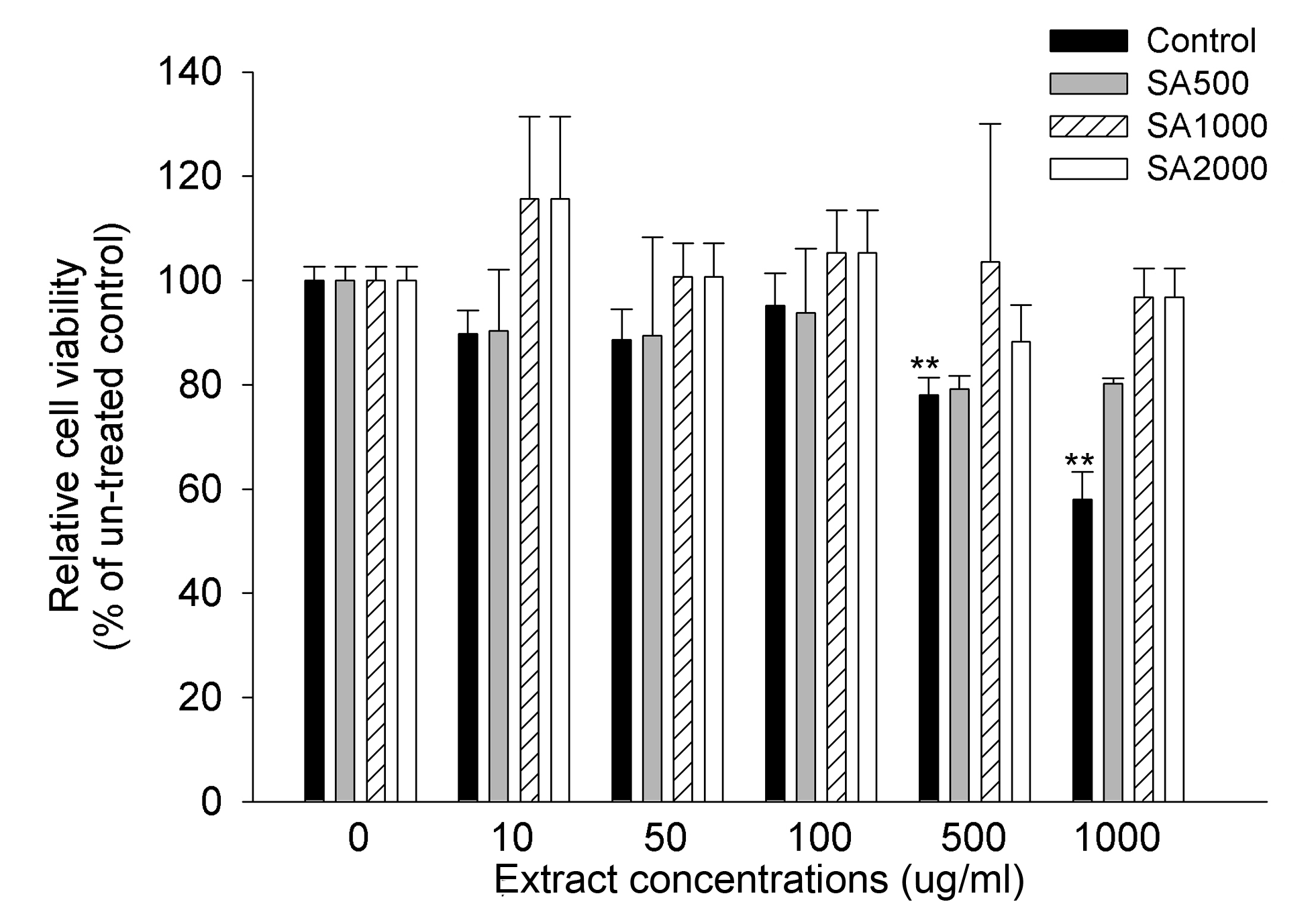

Supplement: Figure S6 — Effect of extracts obtained from elicitor-treated adventitious roots on JB6 P+ cell viability. Extracts were obtained from Aloe vera adventitious roots untreated (Control) or treated with 500 µM SA, 1000 µM SA, or 2000 µM SA. JB6 P+ cells were treated with the indicated amounts of each extract for 4 h, and then 20 µL CellTiter 96 Aqueous One solution was added to the cells and they were incubated for an additional 4 h. Cell viability was subsequently measured at 492 and 690 nm. Data are represented as means of replicate samples ± standard deviation. Statistical analysis was carried out using the Tukey test (* p<0.05, ** p<0.01). Asterisks indicate significant differences compared to control groups. (TIF) [file pone.0082479.s006.tif]
