# Supplementary material for: Enhancement of Anti-Inflammatory Activity of Aloe vera Adventitious Root Extracts through the Alteration of Primary and Secondary Metabolites via Salicylic Acid Elicitation
Source: PLoS One. 2013 Dec 16;8(12):e82479. doi: 10.1371/journal.pone.0082479 (PMC3865001; doi:10.1371/journal.pone.0082479)
Supplement: Table S1 — Primer sets used in this study. (DOCX) [file pone.0082479.s007.docx]

**Table S1. Primer sets used in this study**

|  | Primer | Sequence |
| --- | --- | --- |
| Gene cloning | OKS F | 5’-ATGAGTTCACTCTCCAACGC-3’ |
|  | OKS R | 5’-TCACATGAGAGGCAGGCTG-3’ |
|  | PKS4 F | 5’-ATGAGTTCACTCTCCAACTCTC-3’ |
|  | PKS4 R | 5’-TTACATGAGAGGCAGGCTGTGA-3’ |
| Real-time PCR | OKS realtime primer F | 5’-GGCGGGTCTGATGTTCTAC-3’ |
|  | OKS realtime primer R | 5’-CGAAACTTCTCGGGACGA-3’ |
|  | OKSL-1 realtime primer F | 5’-AGATGGAACCGCAACAGT-3’ |
|  | OKSL-1 realtime primer R | 5’-TGGGAAACTTCTTGAGGAAT-3’ |
|  | OKSL-2 realtime primer F | 5’-CGTCTGCAAGAAAACGATGA-3’ |
|  | OKSL-2 realtime primer R | 5’-CAGAAGACAAGGTGGGTGGT-3’ |
|  | Ubiquitin F | 5’-GGTGGAGTCTTCGGATACCA-3’ |
|  | Ubiquitin R | 5’-TGCTCTCCTTCTGGATGTTG-3’ |
