# Supplementary material for: Enhancement of Anti-Inflammatory Activity of Aloe vera Adventitious Root Extracts through the Alteration of Primary and Secondary Metabolites via Salicylic Acid Elicitation
Source: PLoS One. 2013 Dec 16;8(12):e82479. doi: 10.1371/journal.pone.0082479 (PMC3865001; doi:10.1371/journal.pone.0082479)
Supplement: Table S2 — Effect of plant hormones and media on growth of Aloe vera adventitious roots and accumulation of aloe emodin and chrysophanol after 35 days. (DOCX) [file pone.0082479.s008.docx]

**Table S2. Effect of plant hormones and media on growth of *Aloe vera* adventitious roots and** **accumulation of aloe emodin and chrysophanol after 35 days**

| Plant growth condition (mg/L) | Growth ratio^a^ | Aloe emodin (μg/g)^a^ | Chrysophanol (μg/g)^a^ |
| --- | --- | --- | --- |
|  |  |  |  |
| 0.1 IAA | 0.79±0.11b | 3.08±0.63ab | 34.93±14.02a |
| 0.3 IAA | 1.32±0.11ab | 2.99±0.27ab | 46.63±0.35ab |
| 0.5 IAA | 1.38±0.36ab | 2.36±0.39a | 33.36±0.74a |
| 0.1 IBA | 1.12±0.34ab | 1.69±0.04a | 35.73±0.92a |
| 0.3 IBA | 2.42±0.93a | 4.42±0.54b | 63.65±1.28b |
| 0.5 IBA | 0.99±0.61b | 2.25±0.31a | 37.52±0.97a |
| 0.3IBA+MS | 2.44±0.52b | 3.59±1.92a | 45.07±3.51a |
| 0.3IBA+1/2MS | 1.16±0.46a | 1.39±0.29a | 18.34±0.60b |
| 0.3IBA+2MS | 0.71±0.28a | 3.05±0.17a | 42.12±7.97a |
| 0.3IBA+B5 | 1.31±0.42ab | 2.39±1.10a | 38.99±5.78a |
| 0.3IBA+SH | 1.03±0.40a | 2.30±0.91a | 36.77±0.58a |

^a^ The different characters in each column mean significantly differences (P<0.05) according to LSD test
