# Supplementary material for: Enhancement of Anti-Inflammatory Activity of Aloe vera Adventitious Root Extracts through the Alteration of Primary and Secondary Metabolites via Salicylic Acid Elicitation
Source: PLoS One. 2013 Dec 16;8(12):e82479. doi: 10.1371/journal.pone.0082479 (PMC3865001; doi:10.1371/journal.pone.0082479)
Supplement: Table S3 — Alteration of primary metabolites in Aloe vera adventitious roots following SA treatment. (DOCX) [file pone.0082479.s009.docx]

**Table S3. Alteration of primary metabolites in *Aloe vera* adventitious roots following SA treatment**

|  |  |  | Fold change (Relative to control) | | |
| --- | --- | --- | --- | --- | --- |
| Class | Retention time | Compounds | SA500 | SA1000 | SA2000 |
| Amino acids | 13.3 | L-alanine^ab^ | 0.73 | 1.02 | 0.75 |
|  | 17.0 | L-valine^ab^ | 0.43 | 1.10 | 1.04 |
|  | 19.4 | glycine^ab^ | 0.45 | 0.84 | 0.79 |
|  | 21.1 | L-serine^ab^ | 1.08 | 1.20 | 0.46 |
|  | 21.6 | L-threonine^ab^ | 0.70 | 1.06 | 0.68 |
|  | 25.1 | L-proline^ab^ | 1.46 | 1.95 | 0.99 |
| Carboxylic acids | 24.3 | malic acid^ab^ | 1.30 | 1.64 | 0.88 |
| Sugar alcohols | 25.5 | threitol^ab^ | 0.63 | 1.14 | 0.57 |
|  | 37.2 | myo-inositol^ab^ | 0.77 | 1.18 | 0.71 |
| Sugars | 32.1 | D-xylose^ab^ | 6.72 | 11.19 | 1.05 |
|  | 33.2 | D-ribose^ab^ | 0.87 | 1.07 | 1.03 |
|  | 33.3 | D-fructose^ab^ | 0.83 | 1.08 | 1.03 |
|  | 33.4 | D-glucose^ab^ | 1.87 | 2.47 | 0.97 |
|  | 33.5 | galactose oxime^ab^ | 1.14 | 1.33 | 1.26 |
|  | 34.3 | mannose^ab^ | 0.92 | 1.25 | 0.57 |
|  | 34.5 | mannoonic acid^ab^ | ND | 2.43 | 1.71 |
|  | 36.2 | D-ribofuranoside^ab^ | 0.74 | 0.85 | 1.04 |
|  | 39.3 | D-mannopyranose^ab^ | 0.84 | 1.13 | 0.39 |
|  | 49.2 | melibiose^ab^ | 0.68 | 1.49 | 0.80 |
|  | 50.0 | maltose^ab^ | 0.79 | 1.57 | 0.58 |
|  | 44.3 | unknown^b^ | 3.10 | 1.98 | 1.35 |
|  | 46.4 | unknown^b^ | 0.85 | 1.19 | 0.60 |
|  | 47.2 | unknown^b^ | 0.75 | 1.13 | 0.47 |
|  | 47.4 | unknown^b^ | 0.72 | 1.01 | 0.50 |
|  | 50.2 | unknown^b^ | 0.68 | 1.86 | 0.26 |
|  | 56.0 | unknown^b^ | 0.82 | 0.95 | 0.58 |
|  | 58.2 | unknown^b^ | 3.41 | 1.27 | 3.10 |
|  | 59.1 | unknown^b^ | 0.33 | 0.37 | 0.57 |
|  | 60.0 | unknown^b^ | 0.26 | 2.58 | 5.02 |
| CoA derivatives | 3.1 | malonyl CoA^c^ | 0.88* | 0.21** | 0.07** |
|  | 3.1 | acetyl CoA^c^ | ND | ND | ND |
|  | 3.0 | succinyl CoA^c^ | ND | ND | ND |

^a^ Identified by NIST Mass Spectral Library;

^b^ Analyzed by GC/MS;

^c^ Analyzed by LC/MS in negative mode

ND indicates ‘not detectable’

^*^ Asterisks indicate significant differences compared to control intensity (Tukey test, p<0.05*, p<0.01**)
