# Supplementary material for: Enhancement of Anti-Inflammatory Activity of Aloe vera Adventitious Root Extracts through the Alteration of Primary and Secondary Metabolites via Salicylic Acid Elicitation
Source: PLoS One. 2013 Dec 16;8(12):e82479. doi: 10.1371/journal.pone.0082479 (PMC3865001; doi:10.1371/journal.pone.0082479)
Supplement: Table S4 — Comparison between amino acid sequences identities of OKS and OKSL-1 from Aloe vera and OKS, PKS4, and PKS5 from Aloe arborescens . (DOCX) [file pone.0082479.s010.docx]

**Table S4. Comparison between amino acid sequences identities of OKS and OKSL-1 from *Aloe vera* and OKS, PKS4, and PKS5 from *Aloe arborescens***

|  | AaOKS | AaPKS4 | AaPKS5 |
| --- | --- | --- | --- |
| **AvOKS** | 401/403 (99%) | 401/403 (99%) | 366/403 (91%) |
| **AvOKSL-1** | 388/401 (97%) | 388/401 (97%) | 363/403 (90%) |
