# Supplementary material for: Enhancement of Anti-Inflammatory Activity of Aloe vera Adventitious Root Extracts through the Alteration of Primary and Secondary Metabolites via Salicylic Acid Elicitation
Source: PLoS One. 2013 Dec 16;8(12):e82479. doi: 10.1371/journal.pone.0082479 (PMC3865001; doi:10.1371/journal.pone.0082479)
Supplement: Table S5 — Statistical parameters of PCA, PLS-DA, and OPLS-DA in positive and negative modes. (DOCX) [file pone.0082479.s011.docx]

**Table S5. Statistical parameters of PCA, PLS-DA, and OPLS-DA in positive and negative modes**

|  |  | Positive mode | | Negative mode | | |  |
| --- | --- | --- | --- | --- | --- | --- | --- |
| Treatment | Model | R^2^X | Q^2^ | | R^2^X | Q^2^ | |
| Control, 500 µM SA, 1000 µM SA, and 2000 µM SA | PCA | 0.44 | 0.10 | | 0.62 | 0.40 | |
|  | PLS-DA | 0.56 | 0.76 | | 0.52 | 0.76 | |
|  | OPLS-DA | 0.68 | 0.51 | | 0.80 | 0.68 | |
| Control, 500 µM SA, and 500 µM MJ | PCA | 0.51 | 0.33 | | 0.53 | 0.33 | |
|  | PLS-DA | 0.56 | 0.87 | | 0.53 | 0.61 | |
|  | OPLS-DA | 0.62 | 0.86 | | 0.53 | 0.60 | |
