# Supplementary material for: Enhancement of Anti-Inflammatory Activity of Aloe vera Adventitious Root Extracts through the Alteration of Primary and Secondary Metabolites via Salicylic Acid Elicitation
Source: PLoS One. 2013 Dec 16;8(12):e82479. doi: 10.1371/journal.pone.0082479 (PMC3865001; doi:10.1371/journal.pone.0082479)
Supplement: Table S6 — Statistically significant peak numbers. (DOCX) [file pone.0082479.s012.docx]

**Table S6. Statistically significant peak numbers**

|  | |  | | Induced peaks^a^ | | |  | |
| --- | --- | --- | --- | --- | --- | --- | --- | --- |
| Mode | Peak detection | | SA500 | | SA1000 | SA2000 | | Reduced peaks^a^ |
| Negative | 634 | | 40 | | 20 | 70 | | 114 |
| Positive | 1850 | | 172 | | 63 | 135 | | 267 |

^a^ Number of induced and reduced peaks is based on comparison with peaks of control groups
